# Sub-Saharan Africa’s international migration constrains its sustainable development under climate change

**DOI:** 10.1007/s11625-022-01116-z

**Published:** 2022-03-18

**Authors:** Qirui Li, Cyrus Samimi

**Affiliations:** 1 Africa Multiple Cluster of Excellence, University of Bayreuth, 95440 Bayreuth, Germany; 2 Climatology Research Group, University of Bayreuth, 95447 Bayreuth, Germany; 3 Bayreuth, Centre of Ecology and Environmental Research, University of Bayreuth, 95448 Bayreuth, Germany

**Keywords:** Sustainable Development Goals, Climate extremes, Migration and development, Adaptation of social-ecological systems, Feedback loop, Impact assessment

## Abstract

Sub-Saharan Africa (SSA) is seen as a region of mass migration and population displacement caused by poverty, violent conflict, and environmental stress. However, empirical evidence is inconclusive regarding how SSA’s international migration progressed and reacted during its march to achieving the Sustainable Development Goals (SDGs). This article attempts to study the patterns and determinants of SSA’s international migration and the cause and effects on sustainable development by developing a Sustainability Index and regression models. We find that international migration was primarily intra-SSA to low-income but high-population-density countries. Along with increased sustainability scores, international migration declined, but emigration rose. Climate extremes tend to affect migration and emigration but not universally. Dry extremes propelled migration, whereas wet extremes had an adverse effect. Hot extremes had an increasing effect but were insignificant. SSA’s international migration was driven by food insecurity, low life expectancy, political instability and violence, high economic growth, unemployment, and urbanisation rates. The probability of emigration was mainly driven by high fertility. SSA’s international migration promoted asylum seeking to Europe with the diversification of origin countries and a motive for economic wellbeing. 1% more migration flow or 1% higher probability of emigration led to a 0.2% increase in asylum seekers from SSA to Europe. Large-scale international migration and recurrent emigration constrained SSA’s sustainable development in political stability, food security, and health, requiring adequate governance and institutions for better migration management and planning towards the SDGs.

**Graphical abstract:**

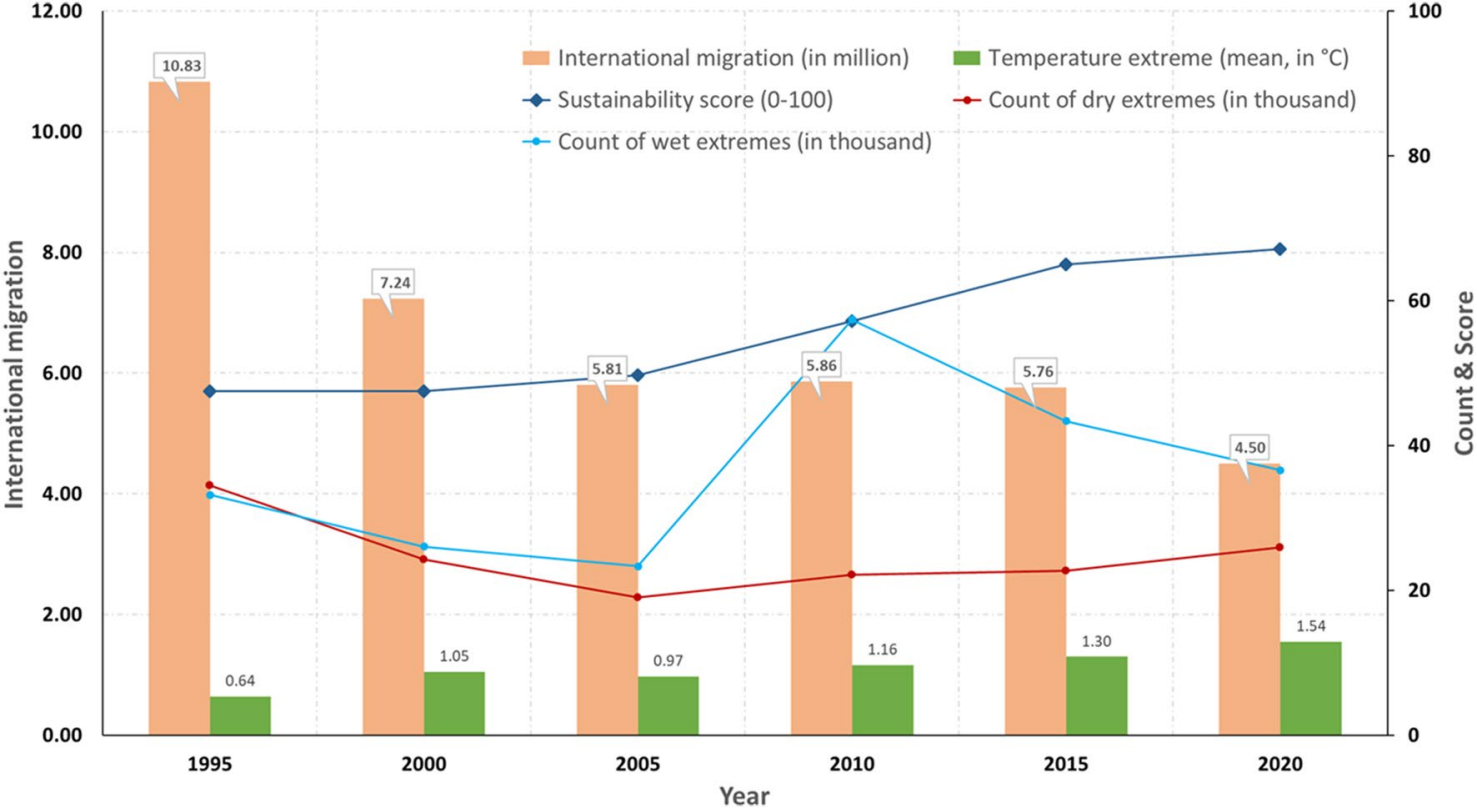

**Supplementary Information:**

The online version contains supplementary material available at 10.1007/s11625-022-01116-z.

## Introduction

Sub-Saharan Africa (SSA) has rich and diverse environmental and demographic potentials and has experienced rapid economic growth over ten years (Ahmed et al. 2016; Jayne et al. 2018). Nevertheless, the region still faces myriad and formidable challenges for sustainable development, like unemployment (Ackah-Baidoo 2016), the lack of health care and education (Appleton et al. 1996), underinvestment in infrastructure (Kodongo and Ojah 2016), debt crisis (Battaile et al. 2015), and the failure of national governance (Ndulu and O’Connell 1999; Davis 2017). As one of the world’s most vulnerable areas to climate change (Niang et al. 2014a; Serdeczny et al. 2017), SSA might get the highest population rise and considerable displaced persons in Africa while making up a significant portion of migrant flows to Europe (van Ittersum et al. 2016; Hoffmann et al. 2020; Cottier and Salehyan 2021). Therefore, scholars and policymakers search for a more positive and proactive migration management process and planning for a better and more sustainable future.

The United Nations adopted the Sustainable Development Goals (SDGs) in 2015 as the developmental agenda for global sustainable development covering 17 goals with 231 indicators that 193 countries have committed to, including SSA (United Nations 2015; United Nations Statistics Division 2017). ‘Orderly, safe, regular and responsible migration’ is a critical issue mentioned in the SDGs, with 11 out of 17 goals being migration related (United Nations Statistics Division 2017; IOM's GMDAC 2019). However, the role of migration in sustainable development and their interactions are still unclear. In addition, migration is influenced by a mix of climatic and environmental, socioeconomic, demographic, cultural, and political factors in the interconnected world (Black et al. 2011). It remains challenging to capture the diverse forms and multiplicities of human migration, monitor and estimate the migration flows and directions, and clarify main migration drivers and predict migration effects on sustainable development (Boas et al. 2019). Reliable data and measurable indicators and theoretical and analytical frameworks are thus needed to integrate interrelated concepts into logical thinking for sophisticated measurement and understanding.

**Fig. 1:**
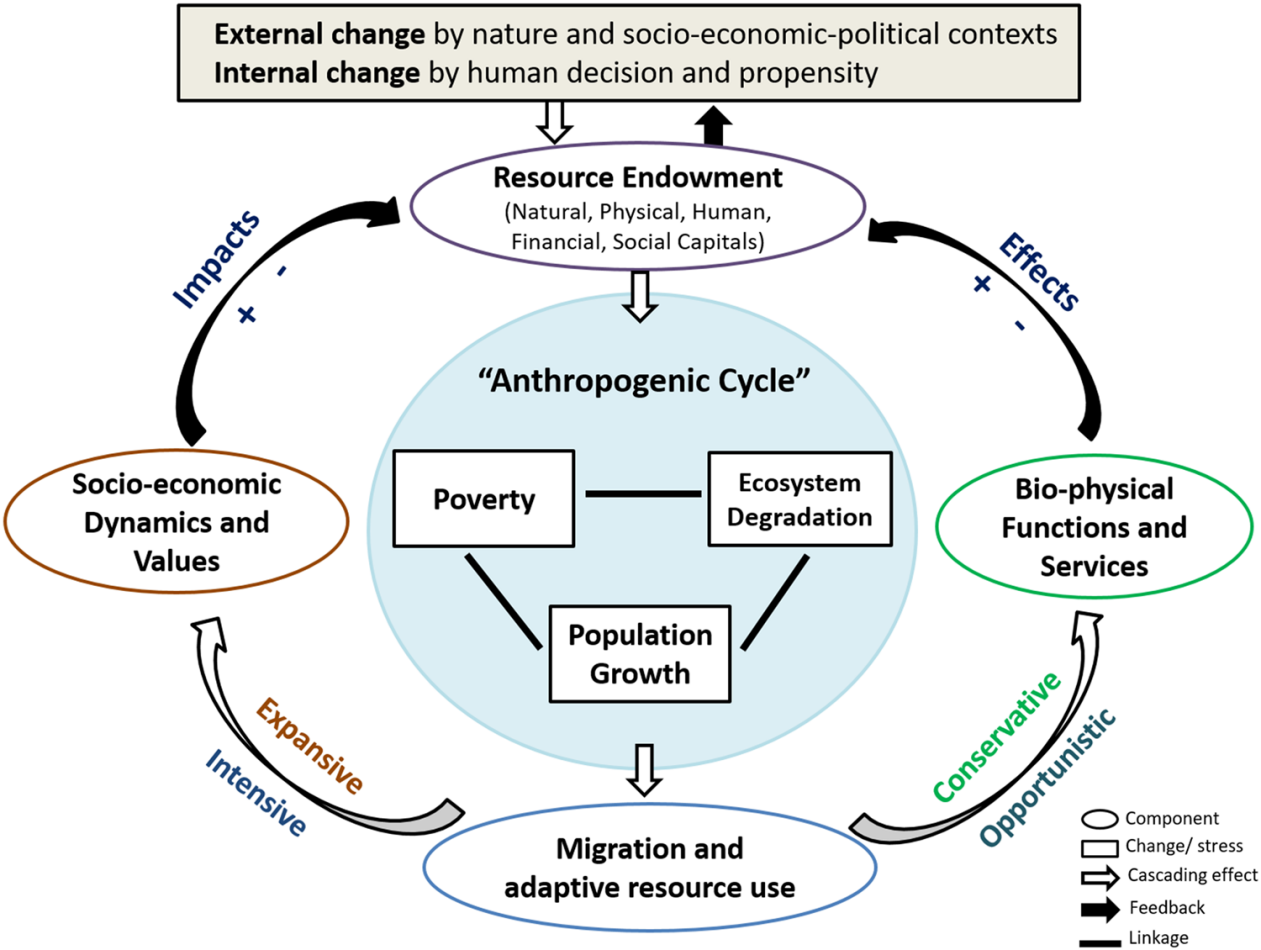
Migration as an adaptation in the feedback loop of coupled social-ecological systems

To fill these gaps, by following the resilience thinking of coupled social-ecological systems (SESs) (Folke et al. 2010, 2016; Folke 2016; Cumming et al. 2017; Marchese et al. 2018), migration is considered a strategy to adapt livelihoods in response to change (Chapin et al. 2010; Bardsley and Hugo 2010; Kniveton et al. 2012; Steffen et al. 2015; Grêt-Regamey et al. 2019; Adger et al. 2020). Change is defined as external and internal by placing people in the centre of analysis (Fig. 1). The internal change is from the difference and variation in human decision and propensity, whereas the external one derives from nature and social-economic-political environments. It indicates that two types of drivers shall at least be considered for the adaptation to change: exogenous and endogenous.

Exogenous drivers usually initiate adaptation when people cannot avoid or prevent the change but conduct actions and strategies to deal with stress and disturbance. It refers more to reactive and autonomous adaptation. By contrast, endogenous drivers come from people's intention to improve their livelihoods, living conditions, environments, and wellbeing. People often take a proactive and planned adaptation to cope with the change and/or manage the system to prevent crisis or disaster. As one form of adaptation, migration depicts the movement of people from one place to another (ex situ) along with their livelihoods and outcomes evolving from one status to another (in situ) over time. Migration is associated with the flow and redistribution of resources, such as human (e.g. labour), financial (e.g. cash and cattle), natural (e.g. land and water), and social (e.g. networks) capitals. Thus, it represents not only individual behaviour (e.g. proactive and reactive adaptation or autonomous-private and public-planned adaptation) (Grothmann and Patt 2005) but also the process of resource allocation or reallocation and utilisation in the entire social-ecological system (Holling 2001; Folke et al. 2010).

In coupled SESs, migration may affect the system state (Higgins 2017) by altering the identity of single agents (or actors) and functional groups as well as the structure (i.e. linkages, relations, and interactions between agents and groups through institutions and infrastructure) and function (i.e. socioeconomic values and bio-physical outputs generated in the process of system development and evolution through various forms of individual and collective activities as well as material exchange and knowledge and information diffusion) of system components. In turn, such dynamics and resultant outcomes, create feedback and fine-tune the external and internal change, exerting further cascading effects within the system and spill-over effects on another system.

Therefore, this paper attempts to assess SSA's international migration patterns and the linkage to sustainable development through observable data and quantitative metrics. It is assumed that SSA countries with varying degrees of resource endowment might have divergent migration patterns, influencing livelihood security and achievements of the SDGs. We acknowledge that our selected indicators cannot comprehensively measure and predict climate-migration-sustainability interlinkages, but quantitative approaches were first illustrated due to the data availability (See details in “Materials and methods”). This work entails a range of questions: (1) What are SSA’s international migration patterns and sustainable development under climate change? (2) What are the determinants of international migration and emigration probability? (3) What are the cascading effects of SSA's international migration on emigrants within SSA and Europe? (4) What are the feedback effects of SSA's international migration on sustainable development? By doing so, this study illustrates new approaches for understanding SSA's international migration patterns and the interlinkage with sustainable development. It paves the way for integrating reliable data and measurable indicators related to migration and the SDGs.

In the remainder of this paper, we first describe materials and methods. An analytical framework shows our study on migration patterns, sustainability assessment, and the environmental and socioeconomic drivers, cascading, and feedback effects of international migration. The empirical analysis explains how we defined migration patterns, composed a sustainability index, and developed models for migration drivers and cascading and feedback effects. After that, we discuss our results and compare them with previous studies. Finally, the conclusions of this research are demonstrated.

## Materials and methods

### Analytical framework

**Fig. 2:**
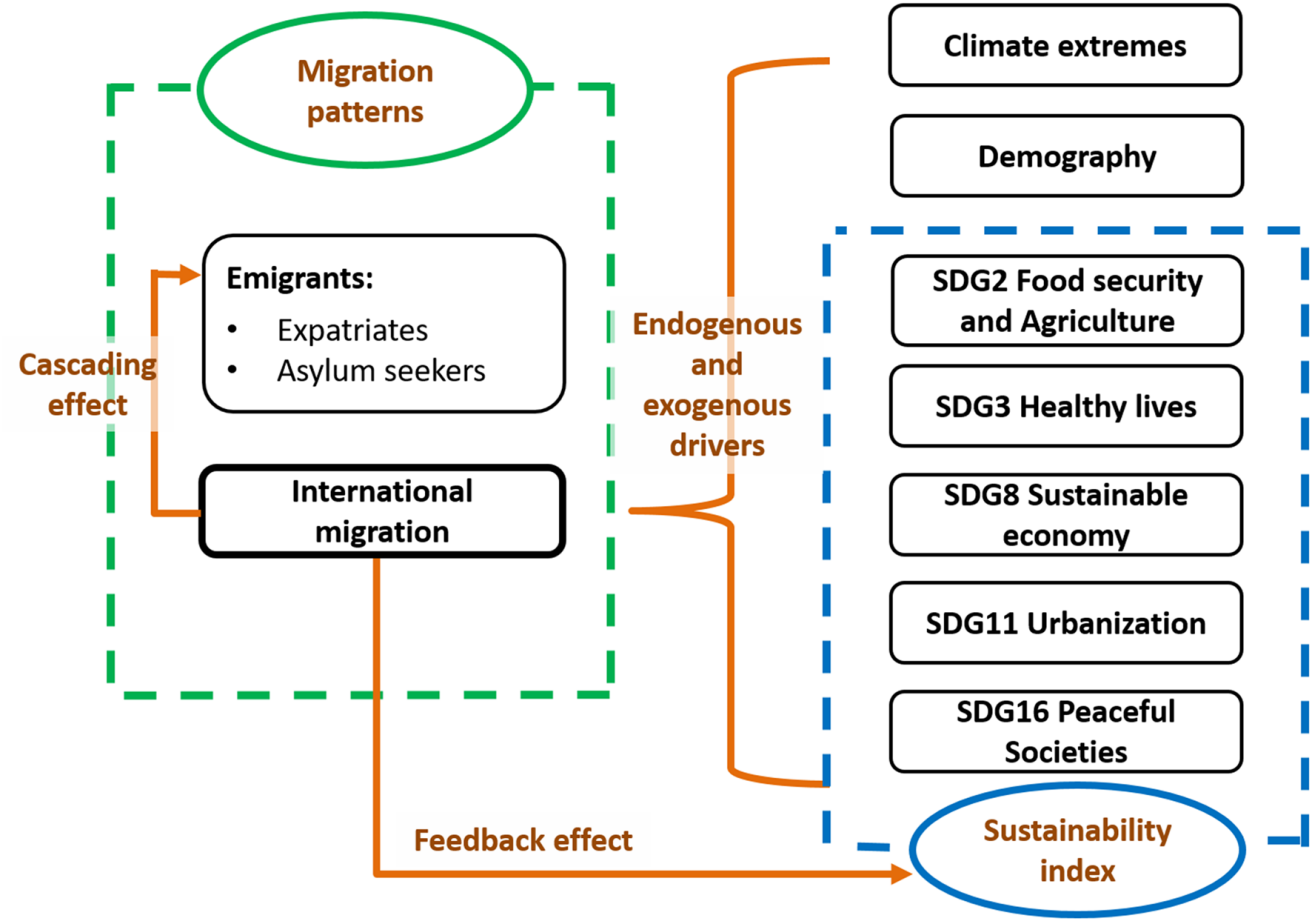
Analytical framework. The framework consists of five analyses: the migration patterns estimate the international migration, expatriates, and asylum seekers of forty SSA countries (Table S1). The sustainability index enables an assessment of sustainability and its five specific goals for those forty countries over the research period. Endogenous and exogenous drivers of SSA's international migration were explored from selected variables concerning climate extremes, demography, and SDG indicators. The cascading effect of SSA's international migration on its emigrants in terms of expatriates and asylum seekers. The feedback effect of international migration on SSA's achievement of the SDGs

According to the research hypotheses and questions, we designed an analytical framework (Fig. 2) which consists of the analysis for international migration patterns, a composite index of sustainability, and regression models for the drivers of international migration and its cascading effects on emigrants and feedback effects on the sustainability index. The framework integrates reliable data from different sources to study SSA’s international migration and sustainable development, providing means to investigate the interlinkage under climatic and demographic changes. It may facilitate measuring and predicting the system adaptation and transformation towards achieving the SDGs through the lens of international migration.

### International migration patterns

As the core of the framework, migration is defined as the adaption of SSA countries to climatic, demographic, and socioeconomic changes. It depicts population movement associated with resource distribution and utilisation, generating various outcomes, and effects on system sustainability. Herein, migration patterns are estimated through the international migration, expatriates, and asylum seekers (Table S1). Although migration is a dynamic process (Diamantides 1994; Massey and Zenteno 1999), we did not consider internal, return, circular, or other forms of migration due to the lack of quality data for SSA countries in time series. It limited our capability to dissect the migratory process dynamics, especially for climate change-related migration (McLeman 2013). Despite this, our model captured the dynamics of international migration regarding the time, place, direction, and circumstances of its occurrence. It derived an unambiguous quantitative measure for investigating the climate-migration-sustainability interlinkages of SSA countries.

International migration is measured by the absolute value of net international migration of an SSA country concerning the latitude (i.e. maximum change) of a coupled system (i.e. an individual SSA country, See Eq. (1)). It depicts the distance of the evolving system from its steady state where the number of immigrants is equal to the number of emigrants (i.e. net migration = 0) throughout the period. A higher value of international migration depicts more changes in the system (or farther from the steady state).

1 $${L}_{\mathrm{it}} =\left|{m}_{I,\mathrm{it}}-{m}_{E,\mathrm{it}}\right|/{D}_{\mathrm{it}}={m}_{\mathrm{it}}/{D}_{\mathrm{it},}$$

where ${L}_{\mathrm{it}}$ is the latitude of a coupled system or country $i$ during time$t$,$m$, ${m}_{I},$ and ${m}_{E}$ represent the international migration, immigration, and emigration, respectively, and the inverse $D$ is the forcing factors that drive population movement and the changes in livelihood outcomes and system status in country$i$. The latitude (i.e. maximum change) can be considered constant for a given system or country at a particular time or moment. Thus, the international migration can be estimated in a reduced form with corresponding coefficients $\beta$ and the error term$\varepsilon$:

2 $$\frac{d{m}_{i}}{dt}=\beta \frac{d{D}_{i}}{dt}+\varepsilon .$$

Although the drivers of international migration differ across countries and contexts, it may take permanent migration into other countries or continents, which changes the number of expatriates and asylum seekers in the receiving countries. This work built a dataset of forty SSA countries (Table S2), referring to data integrity and consistency. The measurement of temporary and return migration is overlooked due to a lack of data.

### Empirical analysis

This study first built a sustainability index of twelve indicators. It is followed by a couple of regression models exploring the environmental and socioeconomic drivers of SSA’s international migration. Another regression model was developed by assuming that SSA's international migration might affect its expatriates in Europe and within SSA and influence its asylum seekers to Europe. Besides, a set of regression modes was composed to estimate the feedback effect of SSA's international migration on sustainable development.

#### Sustainability Index

Indicators are selected for the composite Sustainability Index (Table 1) from the United Nations' Global indicator framework for the Sustainable Development Goals and targets of the 2030 Agenda for Sustainable Development (United Nations 2015; United Nations Statistics Division 2017). According to the description of the SDGs and indicators and the data availability, observable variables were chosen to calculate sustainability scores by taking an equal weighting scheme. We took the mean value of these indicator scores as the goal score if there is more than one indicator under that goal. So does the overall score of sustainability, given that different indicators (or variables) under the same goal (or indicator) reflect different dimensions of sustainability. All scores are transformed into values ranging from 0 to 100. The approach is consistent with the U.N. Sustainable Development Goals reports (https://www.sdgindex.org).

**Table 1:** Descriptive statistics of variables used for the Sustainability Index of sub-Saharan African countries

SDGs	Indicators	Variables	Description	Mean ± standard deviation
**Food security and sustainable agriculture**: Goal 2. End hunger, achieve food security and improved nutrition and promote sustainable agriculture	2.1 Food security: By 2030, end hunger and ensure access by all people to safe, nutritious and sufficient food all year round	Average dietary energy supply adequacy	Dietary Energy Supply (DES) as a percentage of the Average Dietary Energy Requirement (ADER). Each country's or region's average supply of calories for food consumption is normalised by the average dietary energy requirement estimated for its population to provide an index of adequacy of the food supply in terms of calories (Food and Agriculture Organization of the United Nations, Rome, Italy 2020)	103.39 ± 15.83
	2.4 Sustainable agriculture: By 2030, ensure sustainable food production systems and implement resilient agricultural practices	Livestock production index	Net per capita Livestock Production Index Number (2004–2006 = 100)(Food and Agriculture Organization of the United Nations, Rome, Italy 2020)	101.21 ± 16.82
		Crop production index	Net per capita Crop Production Index Number (2004–2006 = 100)(Food and Agriculture Organization of the United Nations, Rome, Italy 2020)	100.17 ± 17.96
		Arable land per capita	Per capita area of arable land (Food and Agriculture Organization of the United Nations, Rome, Italy 2020)	0.23 ± 0.11
		Irrigation share	Share of land area equipped for irrigation in total land area, % (Food and Agriculture Organization of the United Nations, Rome, Italy 2020)	1.42 ± 3.89
**Healthy lives**: Goal 3. Ensure healthy lives and promote wellbeing for all at all ages	Healthy lives: To promote physical and mental health and wellbeing and to extend life expectancy for all	Life expectancy	Average time people in a country are expected to live, based on the year of their birth, in years (United Nations 2019)	55.27 ± 7.05
**Sustainable economy**: Goal 8. Promote sustained, inclusive and sustainable economic growth, full and productive employment and decent work for all	8.1 Economic growth: Sustain per capita economic growth in accordance with national circumstances and achieve higher levels of economic productivity through diversification, technological upgrading and innovation	GDP per capita	Per capita gross domestic product, in current US$ (The World Bank 2020a)	1379.32 ± 2266.34
	8.5 Productive employment: By 2030, achieve full and productive employment and decent work for all women and men	Agro GDP share	Share of agriculture in the total gross domestic product, % (The World Bank 2020a)	24.47 ± 15.31
		Unemployment rate	Share of the labour force without work but available for and seeking employment, % (The World Bank 2020a). (subtractive)	7.24 ± 7.09
**Urbanisation: **Goal 11. Make cities and human settlements inclusive, safe, resilient, and sustainable	11.1 By 2030, ensure access for all to adequate, safe and affordable housing and basic services and upgrade slums	Urbanisation rate	Share of the urban population in the total population of a country, % (The World Bank 2020a)	37.67 ± 16.28
**Peaceful societies:** Goal 16. Promote peaceful and inclusive societies for sustainable development, provide access to justice for all, and build effective, accountable and inclusive institutions at all levels	16.1 Violence and related death: Significantly reduce all forms of violence and related death rates everywhere	Homicide	Rates of homicides per 100,000 population of a country (World Health Organization 2020). (subtractive)	11.82 ± 7.95
		Political stability and absence of violence	Perceptions of the likelihood that the government will be destabilised or overthrown by unconstitutional or violent means, including politically motivated violence and terrorism (The World Bank 2020a)	-0.54 ± 0.88

Descriptions of the Sustainable Development Goals and indicators are adapted from the United Nations’ SDG indicators (United Nations Statistics Division 2017); the number of observations is 240 for forty SSA countries in six periods

In terms of ‘Food security and sustainable agriculture’ in the index, we chose SDG 2 and its indicators 2.1 and 2.4 to guide the measurement via variables concerning dietary energy supply, livestock and crop production, arable land, and irrigation. For ‘Healthy lives’, life expectancy was selected referring to the description of SDG 3. Similarly, the urbanisation rate was chosen to reflect SDG 11.1 on access to adequate housing and essential services in cities. Variables, including per capita gross domestic product (GDP), Agro-GDP share, and unemployment rate, were employed to represent ‘Sustainable economy’ under SDG 8.1 and 8.5. The share of agricultural GDP indicates economic diversification, while the unemployment rate is defined as subtractive. ‘Peaceful societies’ are measured by variables about homicide and political stability under SDG 16.1, where rates of homicides are also defined as subtractive. Data were collected and transformed for those variables by taking the average of every five consecutive years. Missing values were supplemented by alternative data or dismissed with the entire case (or country). Eventually, twelve variables were derived for the Sustainability Index of the forty countries from 1990 to 2018. Although we chose those observable variables referring to the SDGs, the scope and extent are limited by data availability and measuring difficulty. It could be elaborated on in a future study.

#### Drivers of international migration

Based on Eq. (2), a Negative Binomial regression model (NBM) was built, given that the international migration of those forty countries is over-dispersed count data whose conditional variance exceeds its conditional mean. NBM is a generalisation of the Poisson regression model addressing the over-dispersion issue by including a disturbance or error term (see Eq. (3)). We chose exploratory variables by combining climate extremes and demographic drivers with the variables used for the Sustainability Index (Table 2). Previous studies found that positive temperature extremes, rainfall variability, and food insecurity are migration drivers with agriculture as a transmission channel (Mastrorillo et al. 2016; Sadiddin et al. 2019; Carney and Krause 2020). Income opportunities, drought, and violence and armed conflict are found to increase emigration flow (Chort and de la Rupelle 2016; Abel et al. 2019), while the country's living population and fertility, health services, economic growth, and urbanisation play a significant role (Mayda 2010; Castelli 2018).

3 $$\mathrm{log}{m}_{it} ={\psi }_{t}+{\phi }_{i}+{\beta }_{0}+{\beta }_{k}{x}_{ikt{^{\prime}}}+{\sigma \varepsilon }_{it},$$

where ${m}_{it}$ are the expected values of international migration from origin country $i$ at year $t=$ 1995, 2000, 2005, 2010, 2015, and 2020, ${\psi }_{t}$ are time fixed effects, ${\phi }_{i}$ are origin fixed effects, $\beta$ are corresponding regression coefficients, ${\sigma \varepsilon }_{it}$ is the error term, and ${x}_{ik{t}^{^{\prime}}}$ are the values of ${k}^{th}$ exploratory variable for the country $i$ calculated over time intervals ${t}^{^{\prime}}=$ 1990–1994, 1995–1999, 2000–2004, 2005–2009, 2010–2014, and 2015–2018 given endogeneity and reverse causality concerns (except 'life expectancy' that was calculated over time intervals ${t}^{^{\prime}}=$ 1990, 1995, 2000, 2005, 2010, and 2015). The lagged time intervals may help reduce model bias by assuming that exploratory variables are predetermined so that international migration and the error term might only affect their contemporaneous and future values.

**Table 2:** Driving variables of international migration of sub-Saharan African countries

Drivers	Variables	Description	Descriptive		Model 1: International migration from SSA countries		Model 2: International migration from emigration countries		
			Mean	Standard deviation	Incidence rate ratio (IRR)	Coefficients	Emigration selection	Migration coefficients:	
								IRR	Coefficients
Demography	Fertility	Live births per woman represent the average number of live births a hypothetical cohort of women would have at the end of their reproductive period if they were subject during their whole lives to the fertility rates of a given period and if they were not subject to mortality (United Nations 2019)	5.24	1.10	1.2331	0.2095 (0.2062)	6.4584 (1.8245) ^***^	–	–
	Population density	Number of people per square km of land area (United Nations 2019; Food and Agriculture Organization of the United Nations, Rome, Italy 2020)	77.02	89.35	0.9988	− 0.0012 (0.0029)	− 0.0076 (0.0167)	–	–
*Climate change*	Dry extremes	Count of dry extremes (i.e. self-calibrating Palmer Drought Severity Index < -4) within one country of every five-year intervals (van der Schrier et al. 2013; Blunden and Arndt 2020)	618.45	1881.10	1.0001	0.0002 (0.0001)^**^	− 0.0001 (0.0003)	1.0001	0.0001 (0.00004) ^**^
	Wet extremes	Count of wet extremes (self-calibrating Palmer Drought Severity Index > 4) within one country of every five-year intervals (van der Schrier et al. 2013; Blunden and Arndt 2020)	916.35	4131.46	0.9999	− 0.0001 (0.00002)^*^	0.0012 (0.0003) ^***^	0.9999	− 0.0001 (0.00002) ^***^
	Temperature extremes	Maximum value of the FAO temperature change of one country of every five-year intervals, corresponding to the period 1951–1980 (Food and Agriculture Organization of the United Nations, Rome, Italy 2020), in °C	1.11	0.41	1.3470	0.2979 (0.2326)	− 1.0048 (1.5301)	1.1065	0.1012 (0.1526)
Food security and agriculture	Average dietary energy supply adequacy	Dietary Energy Supply (DES) as a percentage of the Average Dietary Energy Requirement (ADER). Each country’s or region’s average supply of calories for food consumption is normalised by the average dietary energy requirement estimated for its population to provide an index of adequacy of the food supply in terms of calories (Food and Agriculture Organization of the United Nations, Rome, Italy 2020)	103.39	15.83	0.9720	− 0.0284 (0.0106)^**^	–	0.9946	− 0.0054 (0.0085)
	Livestock production index	Net per capita Livestock Production Index Number (2004–2006 = 100)	101.21	16.82	0.9995	− 0.0005 (0.0043)	0.1431 (0.0346) ^***^	1.0036	0.0036 (0.0032)
	Crop production index	Net per capita Crop Production Index Number (2004–2006 = 100)	100.17	17.96	0.9973	− 0.0027 (0.0031)	− 0.0052 (0.0174)	0.9898	− 0.0102 (0.0031) ^***^
	Arable land per capita	Per capita area of arable land, in km^2^ per capita	0.23	0.11	0.0161	− 4.1287 (1.1693)^***^	–	0.0172	− 4.0657 (1.2015) ^***^
	Irrigation share	Share of land area equipped for irrigation in total land area, %	1.42	3.89	1.0189	0.0187 (0.0260)	–	1.0241	0.0238 (0.0228)
Healthy lives	Life expectancy	Average time people in a country are expected to live, based on the year of their birth, in years	55.27	7.05	0.9768	− 0.0235 (0.0130)	–	0.9606	− 0.0402 (0.0121) ^***^
Sustainable economy	GDP per capita	Per capita gross domestic product, in current US$	1379.32	2266.34	1.0000	0.00001 (0.00003)	–	1.0003	0.0003 (0.0001) ^**^
	Agro GDP share	Share of agriculture in total gross domestic product, %	24.47	15.31	1.0187	0.0185 (0.0097)	− 0.0125 (0.0459)	0.9994	− 0.0006 (0.0120)
	Unemployment rate	Share of the labour force that is without work but available for and seeking employment, %	7.24	7.09	1.0430	0.0421 (0.0234)	–	1.0797	0.0767 (0.0260) ^**^
Urbanisation	Urbanisation rate	Share of the urban population in the total population of a country, %	37.67	16.30	1.0600	0.0583 (0.0164) ^***^	–	1.0085	0.0085 (0.0152)
Peaceful societies	Homicide	Rates of homicides per 100,000 population of a country	11.82	7.95	1.0266	0.0261 (0.0126) ^*^	–	1.0426	0.0417 (0.0259)
	Political stability and absence of violence	Perceptions of the likelihood that the government will be destabilised or overthrown by unconstitutional or violent means, including politically motivated violence and terrorism	− 0.54	0.88	0.6994	− 0.3575 (0.1067) ^***^	–	0.5274	− 0.6398 (0.0963) ^***^
IMR	Inverse of Mills' ratio	Ratio of the standard normal density divided by the standard normal cumulative distribution function			–	–	–	0.5130	− 0.6675 (0.2265) ^**^
Year fixed effect						Yes	Yes	Yes	
Origin fixed effect						Yes	Yes	Yes	
Constant					100.45	4.6096 (2.0660) ^*^	-83.2790 (16.5590) ^***^	182.3596	5.2060 (1.4286) ^***^
Pseudo *R*^2^ (Nagelkerke)						0.9752	0.7802		0.9986
Count (*N*)						240	240	167	

–, ^*^, ^**^, ^***^ = 0.1, 0.05, 0.01, and 0.001 levels of significance, respectively; figures in parenthesis indicate robust standard errors; *N* depicts the number of observations

As described above, international migration in this work is the absolute value of net international migration to reflect the distance of an evolving system from its steady state. Nevertheless, it shows that those forty countries (Table S2) can be defined as immigration counties (i.e. net migration > 0, e.g. South Africa) and emigration countries (i.e. net migration < 0, e.g. Zimbabwe). They vary over time and have significant differences in their demographic, socioeconomic, and climatic conditions (Table S3). Emigration countries take the majority in SSA, including twenty-nine out of the forty countries, accounting for 68% of the total international migration. It is hence necessary to study the determinants of emigration probability and flow. A Heckman Selection model (Table 2) was developed for the censored subset 'emigration countries' of the data addressing the induced non-random selection bias (Heckman 1979). At the first stage, a Logit regression model was used to predict the likelihood of being an emigration country throughout the research period (see Eq. (4)), in the sense that a binary variable was given as a dependent variable that was assigned a value of 1 if the value of net migration is negative and a value of 0 if positive. In the second stage, another NBM was set up to estimate the international migration from those emigration countries (censored subset) in an unbiased way (see Eq. (5)). The approach attempts to give more insights into SSA's international migration drivers.

4 $${E}_{i}^{^{\prime}}=\mathit{ln}\frac{p}{1-p}={\psi }_{t}+{\phi }_{i}+{\beta }_{0}+{\beta }_{m}{X}_{imt{^{\prime}}}+{\varepsilon }_{it,}$$

where ${E}_{i}^{^{\prime}}$ is the likelihood of origin country $i$ predicted to be an emigration country from explanatory variables ${X}_{im{t}^{^{\prime}}}$ that are the values of *m*th exploratory variable for the country $i$ calculated over time intervals ${t}^{^{\prime}}$, *p* is the probability of a negative value of net international migration, ${\psi }_{t}$ are time fixed effects, ${\phi }_{i}$ are origin fixed effects, $\beta$ are corresponding regression coefficients, and ${\varepsilon }_{it}$ is the error term.

5 $$\mathrm{log}{M}_{lt} ={\psi }_{t}+{\phi }_{l}+{\beta }_{0}+{\beta }_{n}{x}_{lnt{^{\prime}}}+IMR+{\sigma \varepsilon }_{lt,}$$

where ${M}_{lt}$ are the expected values of international migration from emigration country $l (l<i)$ at year $t$ from lagged explanatory variables ${x}_{ln{t}^{^{\prime}}}$ that are the values of *n*th exploratory variable for the country $l$ calculated over time intervals ${t}^{^{\prime}}$, $IMR$ is the inverse Mills' ratio derived from the standard normal and cumulative density functions of Eq. (4), ${\psi }_{t}$ are time fixed effects, ${\phi }_{i}$ are origin fixed effects, ${\beta }_{n}$ are corresponding regression coefficients, and ${\sigma \varepsilon }_{lt}$ is the error term. The model coefficients were interpreted as incidence rate ratios (UCLA: Statistical Consulting Group 2006) and NBM coefficients. Moreover, robust standard errors were taken to obtain unbiased standard errors of coefficients under heteroscedasticity.

#### Cascading effects

SSA's international migration may affect its expatriates (See definition in Table S1) in Europe and within SSA and make up a significant portion of asylum seekers to Europe. Hence, a set of Tobit regression models (Table 3) was employed to estimate the cascading effects given the right censoring (≥ 0) in expatriates and asylum seekers from SSA countries to destination countries. The following equation is given for two separate studies on the cascading effects on expatriates and asylum seekers:

6 $${Y}_{idt}={\psi }_{t}+{\phi }_{i}+{\mathcal{V}}_{d}+\alpha +\beta {x}_{id({t}^{^{\prime}}-1)}+{\gamma m}_{i(t-1)}+{\lambda E}_{i(t-1)}+{\varepsilon }_{idt},$$

where ${Y}_{idt}$ are the expected values of expatriates and asylum seekers from SSA country $i$ to destination country $d$ at year $t=$ 2000, 2005, 2010, 2015, and 2019 for expatriates and $t=$ 2005, 2010, and 2015 for asylum seekers, respectively. ${\psi }_{t}$ are time fixed effects, ${\phi }_{i}$ are origin fixed effects, ${\mathcal{V}}_{d}$ are destination fixed effects, $\alpha , \beta ,$ and γ are corresponding regression coefficients, and ${\varepsilon }_{idt}$ is the error term. ${x}_{id{(t}^{^{\prime}}-1)}$ is a vector of dyadic exploratory variables representing the differential and connection between the origin $i$ and destination $d$ in geographical, demographic, cultural, and socioeconomic factors over time intervals (${t}^{^{\prime}}-1).$ Given data consistency and endogeneity concerns, exploratory variables took the lagged values at ${(t}^{^{\prime}}-1)=$ 1990–1994, 1995–1999, 2000–2004, 2005–2009, and 2010–2014 for expatriates and ${(t}^{^{\prime}}-1)=$ 1995–1999, 2000–2004, and 2005–2009 for asylum seekers, respectively. Previous studies claimed that bilateral migration flows are affected by the growing population, geographical distance, a common land border and language, networks, and per capita GDP (Chort and de la Rupelle 2016; Abel et al. 2019). The population-density differential (Table 3) was used to measure agglomeration and the resultant effect of population growth. Great-circle distance and a dummy variable for land border sharing were included to capture the geographic information between origins and destinations. A dummy variable for colonial language was added to reflect the cultural and colonial ties. We chose historical migrants and migrant ratio to represent social foundations due to the cost-alleviating effect and leading role of networks in international migration (Massey 1988, 1990; Rockenbauch and Sakdapolrak 2017) and the increasing diversification of migration origins and motives (Garcés-Mascareñas and Penninx 2016). Differentials of urbanisation rates and per capita GDP between origins and destinations were added to represent socioeconomic development. Urban and economic growth may increase migration in the short run but gradually eliminate the incentives for movement in the long term (Massey 1988), exerting variant effects on the impetus of migration. ${m}_{i(t-1)}$ is a vector of variables computed for the international migration flow from origin country $i$ at year $\left(t-1\right)$= 1995, 2000, 2005, 2010, and 2015 for expatriates and $\left(t-1\right)$= 2000, 2005, and 2010 for asylum seekers, respectively. A dummy variable ${E}_{i(t-1)}$ was given for origin country $i$ being an emigration country at year $\left(t-1\right)$.

**Table 3:** Marginal effects of international migration on sub-Saharan African expatriates and asylum seekers

Variables	Description	Descriptive		Expatriates in:		Asylum seekers in EU-14 countries
		Mean	Standard deviation	EU-14 countries	Sub-Saharan Africa	
Population density	Differential of population density between an origin country and the destination country, in capita per km^2^	22.91	131.17	− 0.0116 (0.0073)	0.0412 (0.0195)^***^	0.0080 (0.0174)
Distance	Great-circle distance between an origin country to the destination country, in km	3,925,599	2,139,808	− 1.3352 (0.2354)^***^	− 0.4737 (0.1046)^***^	0.3756 (0.5493)
Language	Origin country has the same colonial language as the destination country or not, 1/0 (Exploring Africa 2021)	0.32	0.47	0.0499 (0.0635)	0.1251 (0.0888)^**^	1.2630 (0.1588) ^***^
Border sharing	Origin country shares its land border with the destination country or not, 1/0	0.07	0.25	–	− 0.5990 (0.1623)^***^	–
Historical migrants	Number of the migrant stock originated from SSA in the destination country in 1990	5042.93	39,881.96	0.9339 ( 0.0140)^***^	0.6005 (0.0136)^***^	0.4344 (0.0320) ^***^
Migrant ratio	Ratio of the migrant stock originated from an SSA country to the total migrant stock of the destination country in 1990	0.01	0.07	− 2.0554 (0.9553)^*^	− 2.1974 (0.4608)^***^	− 9.8029 (2.0457) ^***^
Urbanisation	Differential of urbanisation rate between an origin country and the destination country	10.27	27.25	− 0.0399 (0.0232)	0.0062 (0.0299)	− 0.0132 (0.0499)
GDP	Differential of per capita GDP (PPP) between an origin country and the destination country, in current US$	7974.53	14,976.52	− 0.0825 (0.1915)	− 0.0021 (0.0094)	2.3217 (0.5709) ^***^
Emigration country	A country with a negative value of net international migration (i.e. permanent movement of people from one country to another) or not, 1/0	0.69	0.46	− 0.0138 (0.0411)	0.0484 (0.1078)	0.2302(0.1145) ^*^
International migration	Absolute value of net international migration (i.e. the difference between the number of immigrants and the number of emigrants) of an origin country, in thousands	177.47	256.49	0.0108 (0.0247)	0.0113 (0.0447)	0.1877 (0.0553) ^***^
Year fixed effect				Yes	Yes	Yes
Origin fixed effect				Yes	Yes	Yes
Destination fixed effect				Yes	Yes	Yes
McFadden's pseudo-*R*^2^				0.5335	0.5048	0.3452
Count (N)		10,600		2800	7800	1560

– ,^*^, ^**^, ^***^ = 0.1, 0.05, 0.01, and 0.001 levels of significance, respectively; figures in parenthesis indicate robust standard errors; McFadden's values from 0.2 to 0.4 indicate excellent model fit; N depicts the number of observations. The EU-14 grouping includes Austria, Belgium, Denmark, Finland, France, Germany, Greece, Republic of Ireland, Italy, the Netherlands, Portugal, Spain, Sweden, and the United Kingdom

Inverse Hyperbolic Sine transformation was applied for all dependent and exploratory variables to deal with skewness, remaining zero values, and to avoid stacking and disproportionate misrepresentation, given the unique properties of international migration. Marginal effects were presented to explain how dependent variables change when a specific exploratory variable changes while other covariates are constant. Robust standard errors were taken to obtain unbiased standard errors of coefficients under heteroscedasticity.

#### Feedback effects

A set of Ordinary Least Squares regression models (Table 4) was developed to estimate the feedback effect of SSA's international migration on sustainable development. The calculated sustainability scores were estimated in the following equation:

**Table 4:** Effects of international migration on sustainability indexes

Variables	Sustainability indexes					
	Overall score	Food security and agriculture: SDG2	Healthy lives: SDG3	Sustainable economy: SDG8	Urbanisation: SDG11	Peaceful societies: SDG16
Fertility	− 1.5400 (1.9114)	− 9.8620 (3.7251)^**^	2.2640 (1.5275)	0.2253 (1.6490)	0.0454 (1.1449)	− 2.0530 (2.9495)
Population density	− 0.0379 (0.0326)	− 0.0845 (0.0426)^*^	0.0187 (0.0254)	− 0.0529 (0.0194)^**^	− 0.0313 (0.0146)^*^	0.0160 (0.0433)
Dry extremes	− 0.0001 (0.0004)	0.0001 (0.0005)	− 0.00005 (0.0004)	0.0003 (0.0003)	0.00001 (0.0001)	− 0.0006 (0.0007)
Wet extremes	− 0.00005 (0.0001)	0.0001 (0.0001)	− 0.00003 (0.0001)	0.00004 (0.0001)	− 0.00003 (0.00004)	− 0.0001 (0.0002)
Temperature extremes	0.3716 (2.2420)	0.9123 (4.3868)	4.6280 (2.0445)^*^	− 4.9230 (1.9432)^*^	0.6517 (1.1401)	− 2.9990 (2.8833)
Emigration country	− 1.7480 (1.2711)	− 0.6014 (1.9074)	0.1103 (0.9406)	0.2085 (0.9852)	0.1547 (0.5001)	− 5.1420 (1.7727)^**^
International migration	− 0.0071 (0.0028)^*^	− 0.0110 (0.0047)^*^	− 0.0079 (0.0020)^***^	0.0080 (0.0020)^***^	0.0022 (0.0008)^*^	− 0.0119 (0.0042)^**^
Year fixed effect	Yes	Yes	Yes	Yes	Yes	Yes
Origin fixed effect	Yes	Yes	Yes	Yes	Yes	Yes
Adjusted *R*^2^	0.8926	0.5952	0.8834	0.8958	0.9787	0.7781
Constant	61.9810 (15.2560)^***^	112.2200 (27.5060)^***^	35.1980 (11.2960)^**^	50.4180 (12.7910)^***^	52.9790 (8.6561)^***^	72.3630 (23.1940)^**^
Count (*N*)	200	200	200	200	200	200

–, ^*^, ^**^, ^***^ = 0.1, 0.05, 0.01, and 0.001 levels of significance, respectively; figures in parenthesis indicate robust standard errors; *N* depicts the number of observations

7 $${S}_{i{t}^{^{\prime}}}={\psi }_{{t}^{^{\prime}}}+{\phi }_{i}+\alpha +\beta {C}_{i({t}^{^{\prime}}-1)}+{{\omega }_{1}m}_{i \left(t-1\right)}+{{\mu }_{1}E}_{i (t-1)}+{\varepsilon }_{it{^{\prime}}},$$

where ${S}_{it{^{\prime}}}$ are the expected scores of sustainability indexes of country $i$ over time intervals ${t}^{^{\prime}}=$ 1995–1999, 2000–2004, 2005–2009, 2010–2014, and 2015–2018, ${\psi }_{t{^{\prime}}}$ are time fixed effects, ${\phi }_{i}$ are origin fixed effects, $\alpha , \beta , \omega ,$ and μ are corresponding regression coefficients, ${\varepsilon }_{it{^{\prime}}}$ is the error term, ${C}_{i({t}^{^{\prime}}-1)}$ is a vector of control variables concerning climate extremes and demography over time intervals $({t}^{^{\prime}}$–1) = 1990–1994, 1995–1999, 2000–2004, 2005–2009, and 2010–2014, ${m}_{i \left(t-1\right)}$ is the lagged value of international migration at year $\left(t-1\right)$= 1995, 2000, 2005, 2010, and 2015, ${E}_{i (t-1)}$ is the lagged likelihood of country i being an emigration country at year $\left(t-1\right)$. The lagged values guarantee data consistency and reduce model bias given endogeneity and reverse causality issues. Robust standard errors were taken to interpret model results.

### Model validation

Multicollinearity, residual normality, and robustness tests were conducted for the corresponding regression models (See S2 for details).

## Results and discussion

Here, we analysed the dynamics of international migration, expatriates, and asylum seeking among SSA countries, and their progress in sustainable development under climate change from 1995 to 2020. After that, we identified the primary migration drivers regarding climate extremes, food security and agriculture, urbanisation, and peaceful societies, investigated the effect of SSA’s international migration on its expatriates within SSA countries and in EU-14 countries and on its asylum seekers in EU-14 countries, and examined the migration effect on the computed sustainability score and its five aspects (i.e. food security and agriculture (SDG2), healthy lives (SDG3), sustainable economy (SDG8), urbanisation (SDG11), and peaceful societies (SDG16)).

### International migration and sustainable development under climate change

**Fig. 3:**
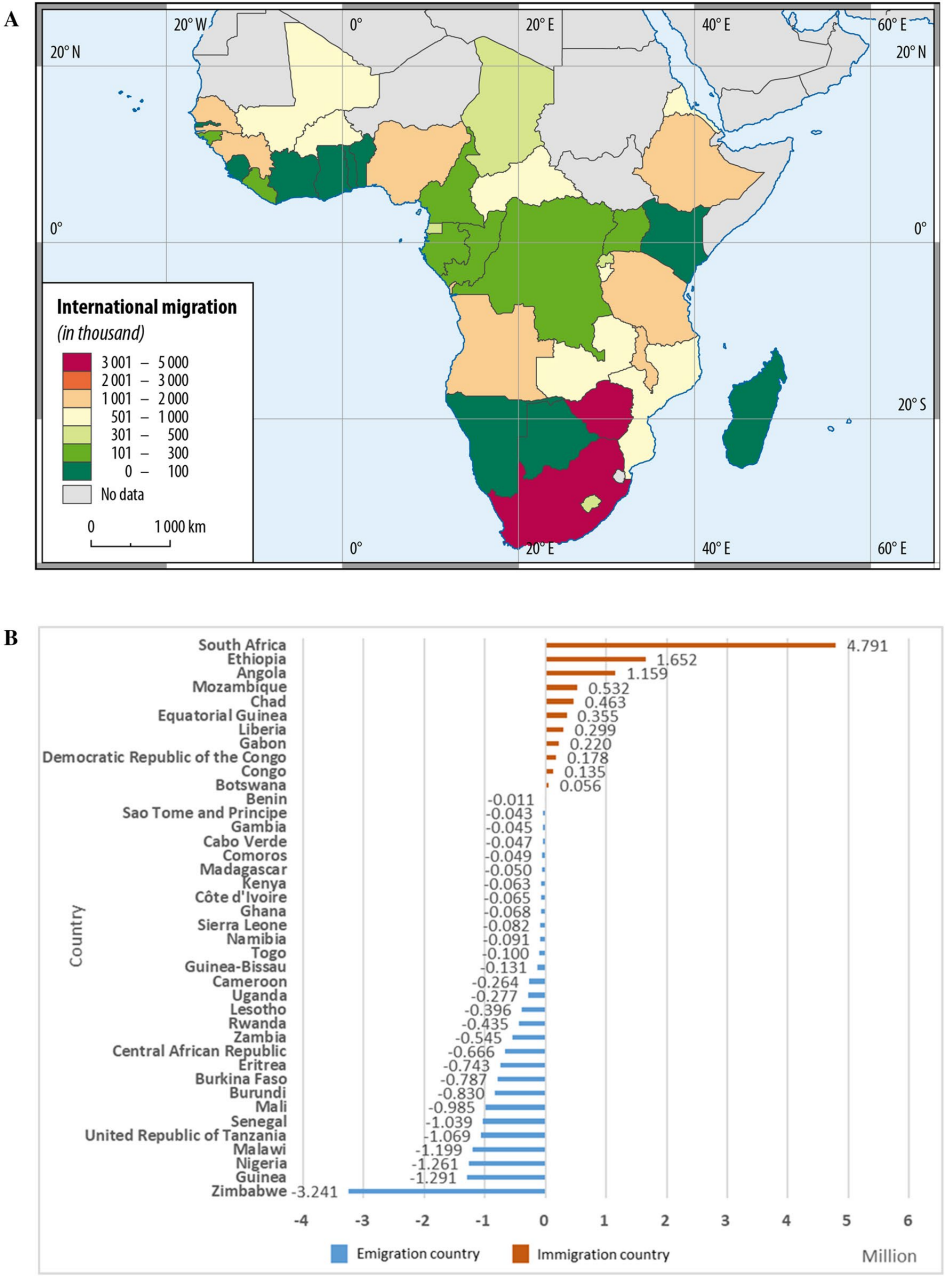

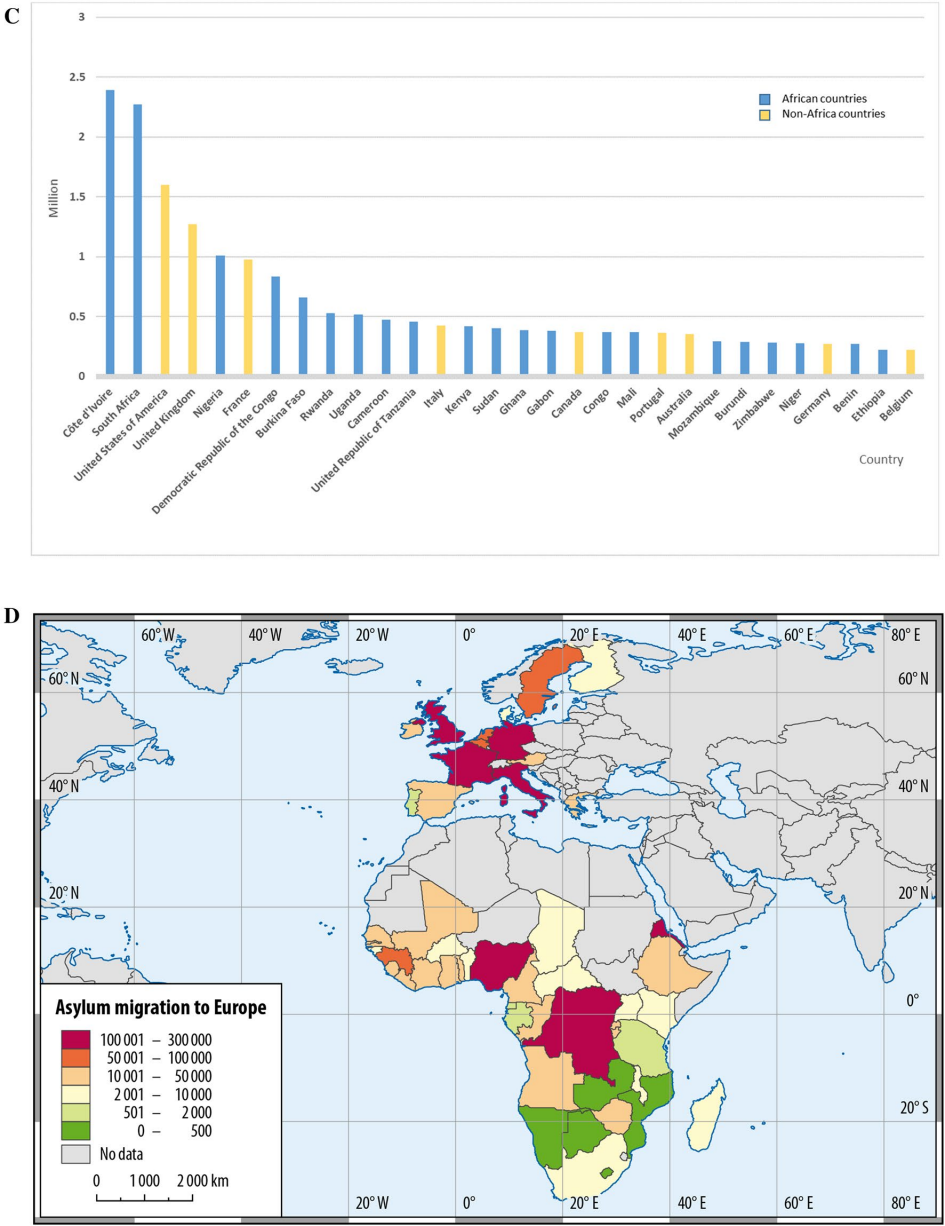
International migration patterns of sub-Saharan Africa. **A** Aggregate international migration from 1995 to 2020. International migration is measured by the absolute value of net international migration (United Nations 2019), reflecting the distance of the evolving system from its steady state (see Eq. (1)). **B** Aggregate net international migration of SSA from 1995 to 2020. A negative value means that people are moving out than moving in and vice versa. **C** Top thirty destination countries for SSA’s expatriates (i.e. migrant stock in Table S1 (United Nations 2020)) in 2019. **D** Aggregate number of SSA's asylum seekers (OECD 2015) in the EU-14 countries from 2001 to 2015. The EU-14 grouping includes Austria, Belgium, Denmark, Finland, France, Germany, Greece, Republic of Ireland, Italy, Netherlands, Portugal, Spain, Sweden, and the United Kingdom

From 1995 to 2020, the international migration from SSA was up to 26 million, with around 6.3 million people moving out. Among those forty countries, South Africa, Zimbabwe, Ethiopia, Guinea, Nigeria, Malawi, Angola, United Republic of Tanzania, Senegal, and Mali were the top ten, which account for 68% of the total SSA migration (Fig. 3A). South Africa, Ethiopia, and Angola were the key immigration countries (Fig. 3B), with a substantial number (about 7.6 million) accounting for 77% of the total immigration flow. The major emigration countries like Zimbabwe, Guinea, Nigeria, Malawi, the United Republic of Tanzania, Senegal, and Mali took up around 63% of the total emigration flow (Fig. 3B). It implies that the hotspots of emigration countries are in West Africa and the junction of South, East, and Central Africa, and that significant immigration countries are of low and lower-middle incomes (World Bank Data Team 2019; The World Bank 2020b). The UN estimates (United Nations 2020) indicate that around 17 of the total 26 million migrants will move within SSA by 2020. Previous studies have also stated that SSA migrants, who represent most African migrants, have moved predominantly within the African continent (Abel and Sander 2014), primarily to low- and middle-income countries (Hoffmann et al. 2020).

International migration may generate changes in SSA expatriates within and outside of Africa (Fig. 3C). The low- and lower-middle-income African countries, like Côte d’Ivoire, South Africa, Nigeria, the Democratic Republic of the Congo, and Burkina Faso, alone accommodated around 7 million SSA migrants. Outside of Africa, the United States of America, the United Kingdom, France, Italy, and Canada were the major destination countries that accommodated 1.6 million, 1.3 million, 979,000, 426,000, and 373,000 people. Besides, part of the outflow may take the form of asylum seeking waves into developed countries. From 2001 to 2015, there were around 1.6 million asylum seekers from SSA to OECD countries. The Horn of Africa, West Africa, and Central Africa (Fig. 3D), comprising Nigeria, Eritrea, the Democratic Republic of the Congo, Guinea, Ethiopia, Mali, Côte d’Ivoire, Gambia, Cameroon, and Zimbabwe, take up almost 71% (OECD 2015). EU-14 countries accommodated 1.2 million people, with Italy, France, Germany, and the United Kingdom alone receiving 56%. Overall, SSA’s international migration seems to be mainly internal to low-and lower-middle-income SSA countries and externally to certain high-income OECD countries. The emigrants are primarily from the Horn of Africa, West Africa, and East-Central Africa. Such international migration patterns underline the complexity and heterogeneity of SSA’s international migration and its linkage to the socioeconomic development of SSA and European societies.

**Fig. 4:**
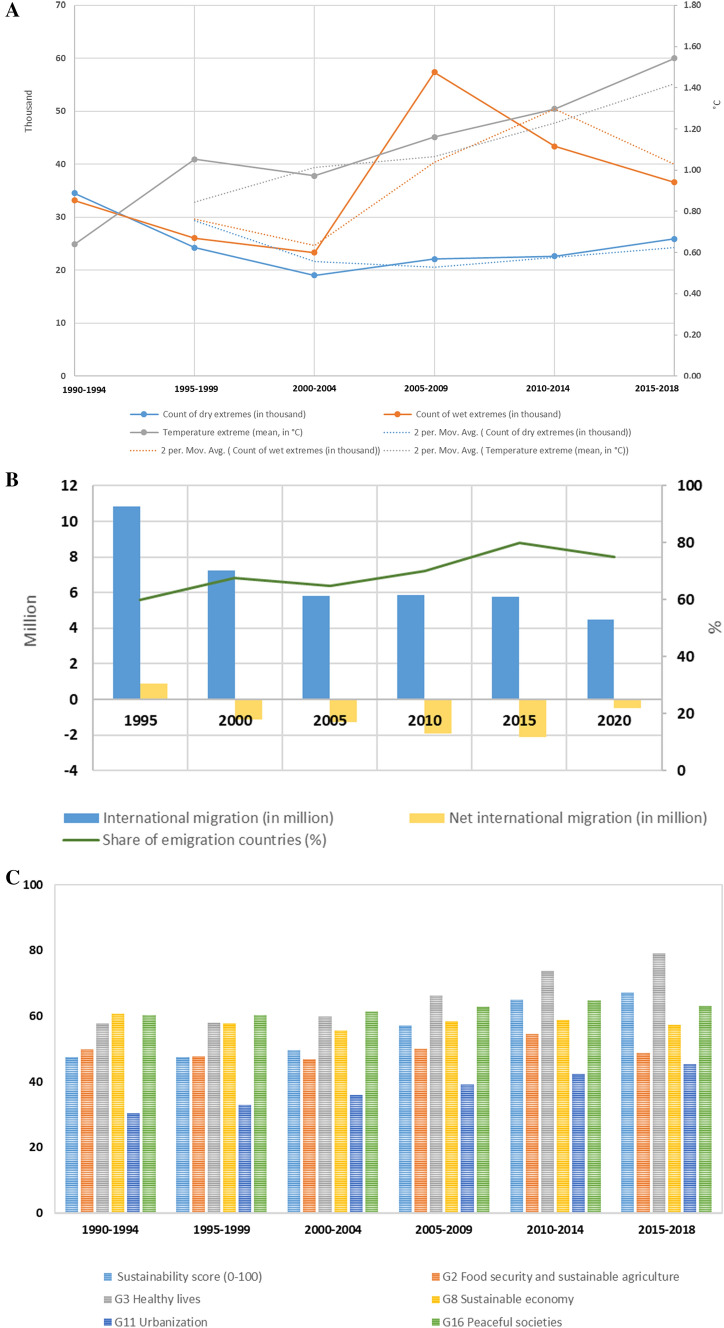

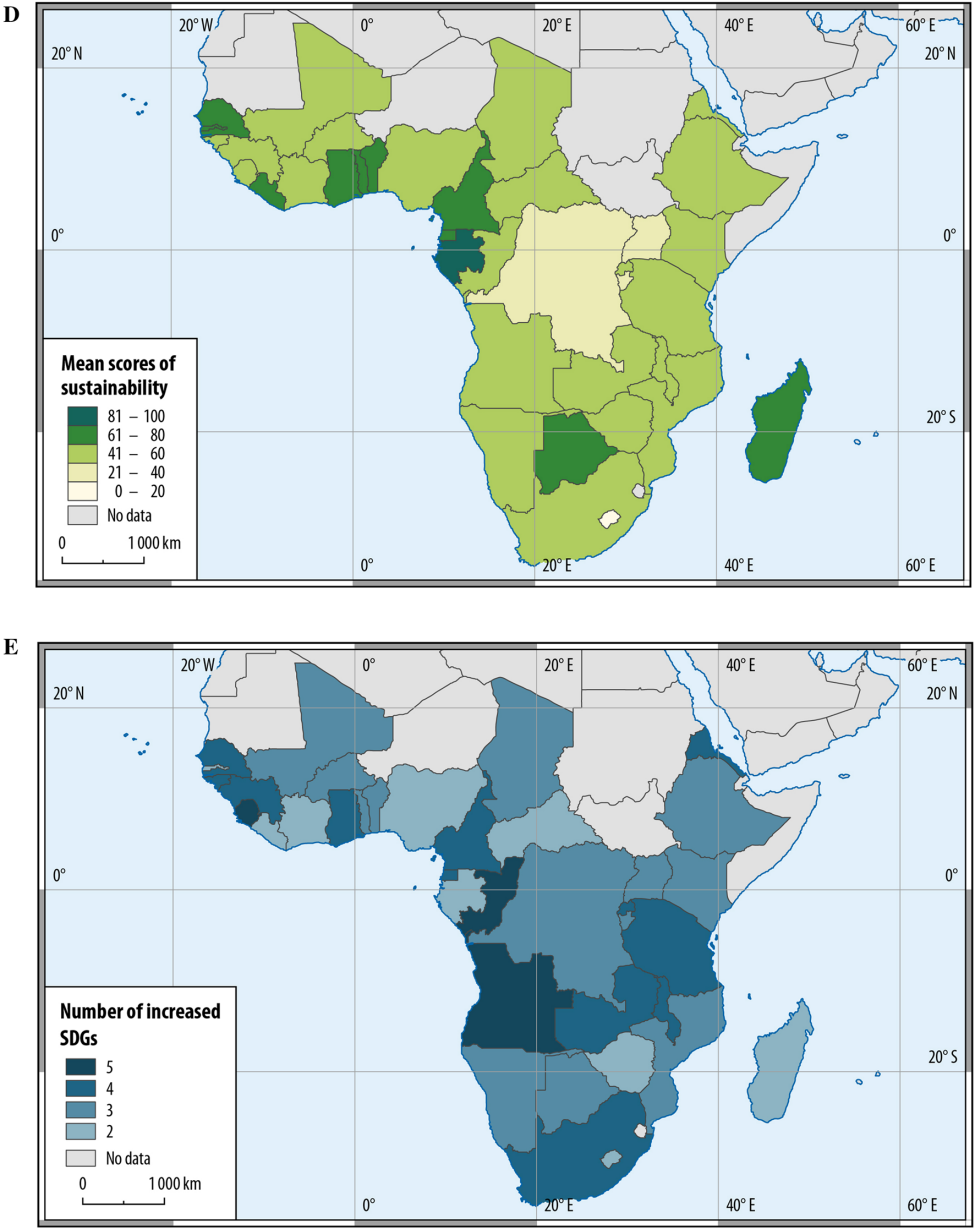
International migration and sustainable development of sub-Saharan Africa under climate change. **A** Climate extremes in SSA countries from 1990 to 2018. Dry and wet extremes count self-calibrating Palmer Drought Severity Index less than − 4 and greater than 4 in a SSA country of every five-year intervals, respectively. Temperature extreme is the maximum value of the FAO temperature change in a SSA country of every five-year interval, corresponding to the reference period 1951–1980. Data sources and descriptions are presented in Table 2. **B** SSA’s international migration from 1995 to 2020. International migration is the absolute value of net international migration (United Nations 2019), reflecting the change of an evolving system from its steady state of population movement (see Eq. (1)). Net international migration is the difference between immigrants and emigrants of each SSA country. Share of emigration countries represents the percentage of SSA countries that had a negative value of net international migration in all forty SSA countries. **C** Sustainability scores of SSA from 1990 to 2018. **D** Mean sore of sustainability calculated based on the SDG indicators for the SSA countries from 1995 to 2018. **E** Number of SDGs with increased scores for each SSA country from 1990 to 2018

Throughout the research period, SSA experienced a slight decline in dry extremes, a wide variability of wet extremes, and a sharp increase in temperature extremes (Fig. 4A). It aligns with the global warming trend, producing more intense and frequent extreme precipitation over West Africa and eastern Africa and more frequent droughts and floods over southern Africa (Niang et al. 2014b; Serdeczny et al. 2017). Southern Africa and the African Sahel are also expected to become warmer and wetter outside the range of their historical year-to-year variability (Mahony and Cannon 2018). Under climate change, SSA’s international migration reduced from about 11 million in 1995 to 4.5 million in 2020. However, it seems that people were increasingly leaving SSA due to the shift of net migration from positive to negative and an increasing share of emigration countries (Fig. 4B).

Meanwhile, there was a significant increase in the score of the Sustainability Index with substantial growth in 'SDG3 healthy lives' (i.e. life expectancy) and 'SDG11 urbanisation' (i.e. urbanisation rates (Fig. 4C). The score of 'SDG16 peaceful societies' slightly rose, whereas 'SDG2 food security and sustainable agriculture' and 'SDG8 sustainable economy' suffered slight decreases. Moreover, West Africa (i.e. Gabon, Ghana and Cameroon) had a higher mean value of the computed sustainability score (Fig. 4D and Table S2). Increases occurred in at least two out of the five computed SDGs and more in southern SSA countries (Fig. 4E) with minor asylum seeking (Fig. 3D). The data demonstrate that the computed sustainability score positively correlated with temperature extremes and negatively correlated with international migration (Table S4 and Figure S1). It also shows significant variations in the international migration, demography, climate extremes, and sustainability indicators across those forty countries over time (Table S5). The results indicate that a higher sustainability score accompanied more significant temperature extremes but less international migration. SSA countries demonstrated different international migration patterns with various degrees of resource endowment and sustainable development under climate warming.

### Drivers of international migration

For SSA's international migration, eleven of the seventeen variables had significant effects, of which seven key drivers (*ρ* value < 0.05) were identified regarding climate extremes, food security and agriculture, urbanisation, and peaceful societies (see Eq. 3 and Model 1 in Table 2). As the primary stress in SSA for agricultural productivity and food security, the results indicate that drought increased international migration. Previous studies have stated that persistent droughts and land degradation threatened food security and aggravated humanitarian conditions while promoting migration in Africa (Gray 2012; Maystadt and Ecker 2014). In contrast, floods and wet extremes had adverse effects on the migration flow. As a new challenge, for local people who lack resources or capacity it would be difficult or even unable for them to make country-to-country moves (Ayeb-Karlsson et al. 2018; Hoffmann et al. 2020). Instead, floods and wet extremes might induce migration within the country. In addition, high temperatures had an insignificant positive effect. It is probably due to the differential effects between middle-income countries and low-income countries and across African regions. Higher temperatures may increase international migration in middle-income countries but decrease the probability in low-income countries (Cattaneo and Peri 2016). Besides, the international migration was stimulated by low dietary energy supply and arable land per capita, high urbanisation and homicide rates, and political instability and violence. Food security has been a big challenge for SSA, pushing populations to move for a sufficient food supply and adequate arable land. The significant urban growth in SSA may promote its international migration not only by attracting immigrants from least-developed countries with rival economies and employment opportunities but also increasing emigration due to associated socioeconomic and environmental issues, like pollution, inadequate infrastructure and services, and crime (Cumming et al. 2014; Li 2020). Civil conflict and violence have also been significant challenges for Africa, affecting livelihood opportunities and propelling migration (Maystadt and Ecker 2014; Kelley et al. 2015; Schleussner et al. 2016).

For SSA's emigration probability (see Eq. 4 and ‘Emigration selection’ of Model 2 in Table 2), a high level of fertility, wet extremes, and livestock production had positive effects. High fertility has been observed in SSA countries for a long time, contributing to the large flow and probability of internal and international migration. The positive effect of wet extremes on SSA's emigration probability is consistent with the wetting trends and increasing emigration in SSA. Wet extremes as a new challenge, especially for vulnerable groups, like females and children, may accelerate the propensity for environmental emigration or asylum seeking from SSA countries. The animal stock in livestock production, often considered savings and wealth, may enable emigration for better living conditions and opportunities, especially under hardship conditions or disasters. Besides, the results about emigration countries (see Eq. 5 and ‘Migration coefficients’ of Model 2 in Table 2) indicate that climate extremes exerted similar effects on the international migration flow. High levels of crop production, arable land per capita, life expectancy, political stability, and the absence of violence deceased the outflow of SSA migrants. In contrast, high GDP per capita and unemployment rates promoted the outflow. It implies that economic growth in SSA may help emigrants afford their emigration costs while people are moving abroad for better employment opportunities and economic wellbeing. In SSA, emigration countries had a smaller migration flow than immigration countries (i.e. IMR coefficient =  − 0.64). It aligns with the above statement that international migration was primarily within SSA.

The results of these two models (Table 2) reveal that climate extremes affected SSA’s international migration, along with population growth, food security, urban and economic growth, and conflict. The effect of climate extremes demonstrated significant differences between the migration flow and direction. For instance, wet extremes decreased SSA’s international migration flow but increased the propensity for emigration. Several studies have also claimed that adverse climatic conditions tended to prompt human displacement and migration but not universally, i.e. in some cases, it reduced migration (Gray 2011, 2012; Mueller and Binder 2015; Challinor et al. 2018). It is thus worth noting that the effect of climate extremes might differ across SSA countries (e.g. low- and middle-income countries), across migration patterns (e.g. internal and international), and between migration flow and direction. The underlying ‘scale issues’ (Eklund et al. 2016) and spatio-temporal processes (Schapendonk et al. 2020) of migration shall be studied in the future.

### Cascading effects of international migration on expatriates and asylum seeking

SSA's international migration had no significant influence on its expatriates within SSA countries nor in EU-14 countries but significantly increased the number of asylum seekers in EU-14 countries (see Eq. 6 and Table 3). 1% more international migration or 1% higher probability of emigration would result in an increase of 0.2% in SSA's asylum seeking to Europe. Historical migrants and the migrant ratio appear to be the most critical drivers. The results indicate that international migration had a small positive effects on SSA's asylum seeking to Europe. In contrast, historical migrants who settled in the destination country before 1990 seemed the primary stimulus to SSA’s expatriates and asylum-seeking growth. It can be explained by network effects that can reduce migration costs and the implementation of visas and other migration restrictions since the 1990s. It also implies that those migration restrictions in Europe might not correspond to less SSA emigration but more asylum seeking and unauthorised migration (Beauchemin et al. 2020). Besides, the negative effect of the migrant ratio and the positive effect of the GDP differential between the EU-14 destination country and SSA origin country convey an increasing diversification of migration origins and motives. The increase in expatriates and asylum seeking appeared to be found more in the SSA country that used to be a minor migrant origin in the destination country. Family reunification might not be the primary motive anymore. Instead, income opportunities and economic wellbeing became the primary driving force for SSA's asylum seeking to Europe. SSA’s international migration diversification might derive from the rapid economic growth since Africa's reforms in the 1990s. The economic growth allowed more SSA people to afford international migration to Europe, Asia, or intra-Africa for security and adequate living conditions (Flahaux and De Haas 2016; Nour et al. 2020).

Moreover, SSA’s expatriates increased along with a short geographical distance between origins and destinations, which often means a shorter travel time and lower migration costs. However, the impact of short distance did not play a significant role in asylum seeking. By contrast, a shared colonial language promoted asylum seeking to EU-14 countries and the expatriates within SSA. These results imply that a common language and relevant cultural and social ties built through a colonial relationship could facilitate the emigration and integration within SSA and Europe. Translocal and transnational social networks that embed people in sending and receiving countries may connect migrants and facilitate the flow of resources, information, and knowledge between places (Rockenbauch and Sakdapolrak 2017; Schapendonk et al. 2020). In addition, the intra-SSA expatriates appeared to be found more in countries with a higher population density and no common land border. Contiguity might contribute more to temporary, internal, or return migration than international migration in SSA, given relevant costs and benefits (Barkin 1967). High population density often means a concentration of economic activities and urban markets which provide job opportunities and attract immigrants. It depicts a manifestation of agglomeration that is often taken as a pathway out of poverty, improving economic returns and social benefits (Fujita et al. 1999; Jacques-François 2000; Borck 2005), especially in less-developed areas, like SSA. Nevertheless, the agglomeration of intra-SSA expatriates might also have adverse effects on sustainable development, in the form of increasing pollution and degradation (e.g. air and soil), pressures on scarce resources (e.g. irrigated farmland and skilled labour), class stratification and inequity (e.g. social exclusion and high-cost of housing), and conflict and violence (e.g. crime and homicide rates). Therefore, SSA’s international migration and resultant agglomeration impacts may influence its achievement of sustainable development.

### Feedback effects of international migration on sustainable development

An increase in SSA's international migration significantly decreased the computed sustainability score (*ρ* < 0.05). The international migration contributed to SDG8 sustainable economy and SDG11 urbanisation but undermined SDG2 food security and agriculture, SDG3 healthy lives, and SDG16 peaceful societies (see Eq. 7 and Table 4). It can be explained by the ‘agglomeration impacts’ of international migration, primarily to low-income but high-population-density countries. The agglomeration may provoke large-scale resource extraction in SSA, leading to land grabbing, competition, and conflict while increasing losses of biodiversity and ecosystem services, climate vulnerability, and trade-offs in the water-food-energy nexus (Biggs et al. 2018). Good governance with efficient institutions and management strategies could enhance positive agglomeration impacts and curtail those negative ones. However, poor governance and the declining institutions facing SSA are formidable, stymieing local capacity building in design and execution while challenging the foundations for sustainable and equitable growth (Sesay 1977; World Bank 1989; United Nations Office on Drugs and Crime 2005).

In SSA, emigration countries gained a lower score of SDG16 peaceful societies (Table 4). SSA has been plagued with political instability, violent conflict, famine, and high crime rates (Adepoju 1995). Recurrent emigration and a significant exodus of skilled labour may exacerbate weak economies and the endemic political instability and violent conflict in countries like Nigeria, Eritrea, and the Democratic Republic of the Congo. Previous studies have claimed violent conflict as an outcome of significant emigration in less-developed areas (Reuveny 2007, 2008) and that migration may adversely affect the political stability of countries (Gebremedhin and Mavisakalyan 2013). In addition, high fertility and population density undermined SDG2 food security and sustainable agriculture. High population density also decreased the scores of SDG8 sustainable economy and SDG11 urbanisation. These results convey that the rapid population growth and agglomeration in SSA increased food demand and pressures on resources and the environment. Since SSA has the lowest cereal self-sufficiency, this may put the region at the most significant food security risk (van Ittersum et al. 2016). The agglomeration of international migration stressed not only food security but also socioeconomic development. Besides, temperature extremes impaired economic sustainability (SDG8) but was associated with an increase in life expectancy (SDG3). The decline of economic growth and productivity can be explained by the wide-ranging negative effect of hot extremes on agricultural output, industrial output, and political stability (Dell et al. 2009, 2012; Moore and Diaz 2015; Burke et al. 2015). From 1990 to 2018, the mean temperature extreme in SSA was around 1.11 °C (Table 2) which was still within the 2 °C catastrophe limit (Huang 2012; Sewe et al. 2018). Although the temperature increase could result in loss of life, SSA still presented significant gains in life expectancy from its continued efforts to improve access to sanitation and clean water and alleviate poverty, malnourishment, and child mortality (Challinor et al. 2018; United Nations 2019; McMaken 2019).

## Conclusion

We conclude that SSA countries demonstrated different international migration patterns with various degrees of resource endowment and sustainable development under climate change. SSA experienced a wide variability of wet extremes and a sharp increase in temperature throughout the research period. Dry extremes increased SSA’s international migration, whereas wet extremes had adverse effects. Temperature extremes had a positive effect but were insignificant, probably due to the differential effects between middle-income countries and low-income countries and across different African regions. The international migration was primarily within SSA to low- and lower-middle-income countries and externally to certain high-income OECD countries. It was driven by low dietary energy supply and arable land per capita, high urbanisation and homicide rates, low political stability, and absence of violence.

Along with the progress in sustainable development, SSA's international migration reduced, but emigration rose in terms of emigrant flows and the number of emigration countries. The probability of emigration was driven by a high level of fertility, wet extremes, and livestock production. In addition to the similar effect of climate extremes mentioned above, the emigration flows were decreased by high crop production, arable land per capita, life expectancy, political stability, and absence of violence but increased by GDP per capita and unemployment rates. SSA’s international migration stimulated asylum seeking in EU-14 countries with the diversification of origin countries and a motive for income opportunities and economic wellbeing. However, the international migration and resultant agglomeration in SSA constrained sustainable development by impairing SDG2 food security and agriculture, SDG3 healthy lives, and SDG16 peaceful societies.

Our work developed a Sustainability Index and regression models that help investigate international migration patterns and drivers, the cascading effects of SSA's international migration on the emigrants within SSA and Europe, and the feedback effects on sustainable development. By using a systematic and evidence-based approach that integrates reliable data, measurable indicators, multidisciplinary concepts, and analytical frameworks, we have provided insights into the feedback loops (Liu et al. 2007) between international migration and sustainable development based on systems thinking, which is not yet well presented in a single migration study. It lays a foundation for further investigating the climate-migration-sustainability interlinkages across multiple dimensions. Nevertheless, internal, return, and circular migration was not considered because of the widespread lack of quality data for SSA countries in time series. It limited the definition of migration and relevant studies in this article. Thus, we call for attention to improving the availability of quality data on migration and ensuring the monitoring of all migrants and migration flows which are essential to improve migration management and policy. Comprehensive and data-available indicators are also required to measure and predict migration propensity, flow, and capacity and the trade-offs and synergies between achieving different SDGs, given the emerging challenges from COVID-19 (Lambert et al. 2020; Forster et al. 2020; Ottersen and Engebretsen 2020).

## Supplementary Information

Below is the link to the electronic supplementary material.

Supplementary file1 (DOCX 591 kb)
