# Supplementary material for: Sub-Saharan Africa’s international migration constrains its sustainable development under climate change
Source: Sustain Sci. 2022 Mar 18;17(5):1873–97. doi: 10.1007/s11625-022-01116-z (PMC8931456; doi:10.1007/s11625-022-01116-z)
Supplement: Supplementary file 1 — Supplementary file1 (DOCX 591 kb) [file 11625_2022_1116_MOESM1_ESM.docx]

**Sub-Saharan Africa’s international migration constrains its sustainable development under climate change**

**Contents of Supplementary Information:**

- Supplementary Tables
- Supplementary Figures
- Model Validation
- Multicollinearity test
- Residual normality test
- Robustness test
- References

# **SUPPLEMENTARY TABLES**

**Table S1. Descriptive statistics of variables used for the migration patterns of sub-Saharan African countries**

| **Indicators** | **Description** | **Mean** | **Standard deviation** |
| --- | --- | --- | --- |
| International migration | Absolute value of the net international migration (i.e. the difference between the number of immigrants and the number of emigrants) from 1995 to 2020 (United Nations 2019), in thousands. | 177.47 | 256.49 |
| Expatriates | Migrant stock, i.e. the number of SSA migrants residing in a country other than their native country from 1995 to 2020 (United Nations 2020). | 5191.34 | 35590.15 |
| Asylum seekers | Number of people have left a country and is seeking protection from persecution and serious human rights violations in an EU-14 country from 2001 to 2015 (OECD 2015). | 599.50 | 2290.80 |

Note: the EU-14 grouping includes Austria, Belgium, Denmark, Finland, France, Germany, Greece, Republic of Ireland, Italy, Netherlands, Portugal, Spain, Sweden, and the United Kingdom; the number of observations is 242 for International Migration, 2800 for Expatriates in EU-14 countries and 7800 for Expatriates in SSA, and 1560 for Asylum Seekers throughout the period.

**Table S2. Migration patterns and sustainability of the selected sub-Saharan African (SSA) countries**

| **Country (N=40)** | **Sustainability Index score** | **Number of SDGs with increased scores** | **Net international migration** | **Expatriates within SSA** | **Expatriates in EU-14** | **Asylum seekers in EU-14** |
| --- | --- | --- | --- | --- | --- | --- |
| Angola | 53.20 | 5 | 1159000 | 652190 | 225466 | 45146 |
| Benin | 75.86 | 3 | -11000 | 666288 | 32101 | 5128 |
| Botswana | 73.00 | 3 | 56000 | 86435 | 4836 | 836 |
| Burkina Faso | 48.04 | 3 | -787000 | 1581023 | 29547 | 13295 |
| Burundi | 28.73 | 3 | -830000 | 623972 | 25034 | 20575 |
| Cabo Verde | 88.86 | 3 | -47000 | 177102 | 114537 | 228 |
| Cameroon | 60.50 | 4 | -264000 | 381591 | 172677 | 56331 |
| Central African Republic | 45.09 | 2 | -666000 | 762947 | 20673 | 7486 |
| Chad | 42.86 | 3 | 463000 | 198494 | 11321 | 9441 |
| Comoros | 60.07 | 3 | -49000 | 60055 | 40728 | 7592 |
| Congo | 59.13 | 5 | 135000 | 238597 | 108574 | 35084 |
| Côte d'Ivoire | 54.79 | 2 | -65000 | 1113492 | 157930 | 64151 |
| Democratic Republic of the Congo | 36.72 | 3 | 178000 | 1592529 | 238496 | 131986 |
| Equatorial Guinea | 76.18 | 4 | 355000 | 125588 | 22820 | 1085 |
| Eritrea | 48.64 | 4 | -743000 | 731228 | 207695 | 250877 |
| Ethiopia | 41.44 | 3 | 1652000 | 834970 | 149937 | 72502 |
| Gabon | 94.07 | 2 | 220000 | 46616 | 25221 | 1330 |
| Gambia | 70.36 | 2 | -45000 | 118417 | 71959 | 59976 |
| Ghana | 73.89 | 4 | -68000 | 967377 | 283092 | 48139 |
| Guinea | 48.11 | 4 | -1291000 | 529871 | 88389 | 89040 |
| Guinea-Bissau | 56.55 | 4 | -131000 | 101238 | 43394 | 9527 |
| Kenya | 46.00 | 3 | -63000 | 512556 | 194397 | 16555 |
| Lesotho | 14.04 | 2 | -396000 | 341541 | 1299 | 54 |
| Liberia | 65.09 | 2 | 299000 | 219263 | 15012 | 23338 |
| Madagascar | 62.16 | 2 | -50000 | 162269 | 144076 | 2884 |
| Malawi | 46.96 | 4 | -1199000 | 324507 | 22506 | 3190 |
| Mali | 56.78 | 3 | -985000 | 1263741 | 122488 | 65966 |
| Mozambique | 54.18 | 3 | 532000 | 1011195 | 85397 | 253 |
| Namibia | 54.07 | 3 | -91000 | 195413 | 4652 | 1693 |
| Nigeria | 44.80 | 2 | -1261000 | 1447028 | 437813 | 263347 |
| Rwanda | 46.00 | 4 | -435000 | 498950 | 34140 | 22024 |
| Sao Tome and Principe | 78.61 | 4 | -43000 | 36891 | 19852 | 14 |
| Senegal | 72.21 | 4 | -1039000 | 647369 | 308340 | 42162 |
| Sierra Leone | 49.60 | 5 | -82000 | 154955 | 45338 | 41129 |
| South Africa | 52.66 | 4 | 4791000 | 847481 | 332932 | 5696 |
| Togo | 63.34 | 3 | -100000 | 530757 | 57969 | 26044 |
| Uganda | 36.21 | 3 | -277000 | 598330 | 99480 | 16595 |
| United Republic of Tanzania | 54.02 | 4 | -1069000 | 322152 | 61976 | 3592 |
| Zambia | 53.56 | 4 | -545000 | 316460 | 82226 | 1014 |
| Zimbabwe | 40.48 | 2 | -3241000 | 940967 | 208360 | 55814 |

Note: Sustainability Index score here is the mean score calculated based on the SDG indicators for the SSA countries from 1995 to 2018; net international migration is the aggregates of the SSA countries from 1995 to 2020, where negative values indicate emigration countries and positive values depict immigration countries; expatriates within SSA and Expatriates in EU-14 take the value of those countries by 2020; asylum seekers in EU-14 is the aggregate from 2001 to 2015.

**Table S3. Differences in demography, socio-economic and climatic conditions between immigration countries and emigration countries in sub-Saharan Africa (mean ± standard deviation)**

| Drivers | Variables | Immigration country (N=11) | Emigration country (N=29) | All (N=40) |  |
| --- | --- | --- | --- | --- | --- |
| Demography | Fertility | 5.24±1.25 | 5.25±1.03 | 5.24±1.1 |  |
|  | Population Density | 47.30±70.80 | 90.01±93.60 | 77.02±89.35^***^ |  |
| Climate change | Dry Extremes | 485.75±1429.51 | 676.46±2048.70 | 618.45±1881.1 |  |
|  | Wet Extremes | 448.27±1930.62 | 1120.96±4777.04 | 916.35±4131.46 **.** |  |
|  | Temperature extremes | 1.01±0.39 | 1.15±0.42 | 1.11±0.41^*^ |  |
| Food security and agriculture | Average dietary energy supply adequacy | 102.26±16.53 | 103.89±15.53 | 103.39±15.83 |  |
|  | Livestock production index | 99.06±14.53 | 102.15±17.68 | 101.21±16.82 |  |
|  | Crop production index | 100.30±21.80 | 100.12±16.07 | 100.17±17.96 |  |
|  | Arable land per capita | 0.23±0.11 | 0.23±0.11 | 0.23±0.11 |  |
|  | Irrigation share | 0.82±2.59 | 1.68±4.32 | 1.42±3.89 |  |
| Healthy lives | Life expectancy | 54.50±5.98 | 55.61± 7.46 | 55.27±7.05 |  |
| Sustainable economy | GDP per capita | 2484.80± 3633.92 | 896.09±946.08 | 1379.32±2266.34 **.** |  |
|  | Agro-GDP share | 21.84±19.17 | 25.61±13.18 | 24.47±15.31^*^ |  |
|  | Unemployment rate | 8.94± 8.47 | 6.49± 6.27 | 7.24±7.09 |  |
| Urbanization | Urbanization rate | 44.34±19.50 | 34.76±13.74 | 37.67±16.28^***^ |  |
| Peaceful societies | Homicide | 13.05±10.65 | 11.28±6.38 | 11.82±7.95 |  |
|  | Political stability and absence of violence | -0.56±0.94 | -0.53± 0.85 | -0.54±0.88 |  |

. , ^*^, ^**^, ^***^ = 0.1, 0.05, 0.01 and 0.001 levels of significance in differences suggested by non-parametric Kruskal–Wallis test. N depicts the number of observed SSA countries, i.e., 240 observations of forty SSA countries, particularly 167 observations for SSA emigration countries from 1990 to 2018.

**Table S4. Kendall's rank correlation matrix**

| Variables | Dry extremes | Wet extremes | Temperature extremes | International migration | Probability of emigration | Sustainability score |
| --- | --- | --- | --- | --- | --- | --- |
| Dry extremes | 1.000 |  |  |  |  |  |
| Wet extremes | 0.310^***^ | 1.000 |  |  |  |  |
| Temperature extremes | -0.079 | 0.025 | 1.000 |  |  |  |
| International migration | -0.041 | 0.020 | -0.100^*^ | 1.000 |  |  |
| Probability of emigration | -0.017 | 0.110 | 0.120^*^ | -0.081 | 1.000 |  |
| Sustainability score | -0.044 | -0.066 | 0.300^***^ | -0.320^***^ | -0.021 | 1.000 |

^*^, ^**^, ^***^ = 0.05, 0.01 and 0.001 levels in significance; 240 observations of 40 countries. The definition of variables is shown in Table 2.

**Table S5. Differences in international migration and its potential drivers**

| **Variables** | **Mean ± standard deviation** | **Difference between 1995 and 2020** | **Difference across forty SSA countries** |
| --- | --- | --- | --- |
| International migration | 166.64 ± 247.59 | 5.290 | 159.940^***^ |
| Fertility | 5.24 ± 1.10 | 65.858^***^ | 155.070^***^ |
| Population density | 77.02 ± 89.35 | 8.343 | 2228.410^***^ |
| Dry extremes | 618.45 ± 1881.10 | 2.069 | 160.340^***^ |
| Wet extremes | 916.35 ± 4131.46 | 3.440 | 142.890^***^ |
| Temperature extremes | 1.11 ± 0.4 | 117.990^***^ | 72.800^***^ |
| Average dietary energy supply adequacy | 103.39 ± 15.83 | 14.679^*^ | 202.810^***^ |
| Livestock production index | 101.21 ± 16.82 | 10.035**.** | 59.097^*^ |
| Crop production index | 100.17 ± 17.96 | 7.878 | 39.679 |
| Arable land per capita | 0.23 ± 0.11 | 12.406^*^ | 207.420^***^ |
| Irrigation share | 1.42 ± 3.89 | 1.567 | 231.550^***^ |
| Life expectancy | 55.27 ± 7.05 | 93.624^***^ | 104.540^***^ |
| GDP per capita | 1379.32 ± 2266.34 | 53.069^***^ | 159.920^***^ |
| Agro GDP share | 24.47 ± 15.31 | 7.078 | 215.190^***^ |
| Unemployment rate | 7.24 ± 7.0 | 3.453 | 214.320^***^ |
| Urbanization rate | 37.67 ± 16.30 | 15.179^**^ | 217.090^***^ |
| Homicide | 11.82 ± 7.95 | 12.309^*^ | 204.200^***^ |
| Political stability and absence of violence | -0.54 ± 0.88 | 0.229 | 190.310^***^ |

**.** , ^*^, ^**^, ^***^ = 0.1, 0.05, 0.01 and 0.001 levels in differences suggested by non-parametric test; The difference is demonstrated in terms of Kruskal-Wallis chi-squared value and p-value across forty SSA countries (Table S2) throughout 1995, 2000, 2005, 2010, 2015 and 2020. The definition of variables is shown in Table 2.

# **SUPPLEMENTARY FIGURES**


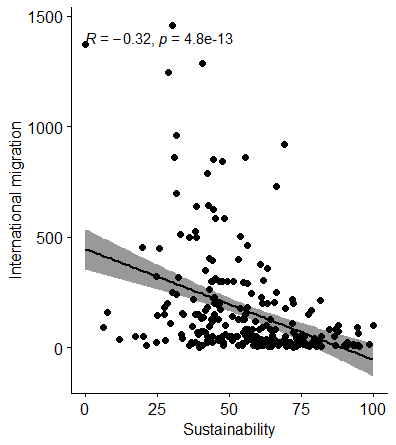


**Figure S1. Kendall's rank correlation between the sustainability score and international migration of the forty SSA countries throughout the period.**

# **MODEL VALIDATION**

## **Multicollinearity test**

Variance inflation factors (VIF) help identify the degree of multicollinearity in the model of international migration drivers (Table 2), cascading effects (Table S7), and feedback effects (Table S8). The value of VIF shall be less than 10.

**Variance inflation factors demonstrated for the model of international migration drivers.**

| **Variables** | | **Model1:** International migration from SSA countries | **Model2:** International migration from emigration countries | |
| --- | --- | --- | --- | --- |
|  |  |  | Emigration selection | Migration coefficients: |
| Fertility | | 5.46 | 7.2 | — |
| Population density | | 5.69 | 6.44 | — |
| Dry extremes | | 2.78 | 2.14 | 2.83 |
| Wet extremes | | 2.45 | 3.62 | 2.78 |
| Temperature extremes | | 2.33 | 2.61 | 2.2 |
| Average dietary energy supply adequacy | | 3.66 | — | 4.93 |
| Livestock production index | | 1.47 | 2.17 | 1.68 |
| Crop production index | | 1.42 | 1.57 | 1.5 |
| Arable land per capita | | 3.97 | — | 4.14 |
| Irrigation share | | 4.84 | — | 4.72 |
| Life expectancy | | 2.73 | — | 2.53 |
| GDP per capita | | 2.44 | — | 3.51 |
| Agro GDP share | | 3.69 | 2.64 | 3.75 |
| Unemployment rate | | 5.23 | — | 4.88 |
| Urbanization rate | | 7.72 | — | 7.56 |
| Homicide | | 4.14 | — | 6.31 |
| Political stability and absence of violence | |  | — | 2.68 |
| Inverse of Mills' ratio |  | — | — | 2.35 |

**Variance inflation factors demonstrated for the model of cascading effects.**

| **Variables** | **Expatriates in:** | | **Asylum Seekers in EU-14 countries** |
| --- | --- | --- | --- |
|  | EU-14 countries | Sub-Saharan Africa |  |
| Population density | 2.48 | 3.76 | 2.59 |
| Distance | 5.52 | 3.26 | 4.63 |
| Language | 1.82 | 1.45 | 1.66 |
| Border sharing | — | 2.2 | — |
| Historical migrants | 2.67 | 2.53 | 2.58 |
| Migrant ratio | 1.32 | 1.71 | 1.35 |
| Urbanization | 2.18 | 4.11 | 2.09 |
| GDP | 6.29 | 2.77 | 6.28 |
| Emigration country | 1.44 | 1.67 | 1.69 |
| International migration | 2.12 | 2.36 | 2.25 |
| Year fixed effect | 1.48 | 1.09 | 2.03 |
| Origin fixed effect | 1.15 | 1.18 | 1.14 |
| Destination fixed effect | 1.42 | 1.19 | 1.37 |

**Variance inflation factors demonstrated for the model of feedback effects.**

| **Variables** | Fertility | Population density | Dry extremes | Wet extremes | Temperature extremes | Emigration country | International  migration | Year fixed effect | Origin fixed effect |
| --- | --- | --- | --- | --- | --- | --- | --- | --- | --- |
| **VIF** | 5.5 | 6.04 | 2.87 | 2.27 | 2.17 | 1.49 | 1.57 | 1.32 | 1.16 |

## **Residual normality test**

Normal Q-Q plots are used to check the residual normality of the model of international migration drivers (Table 2), cascading effects (Table S7), and feedback effects (Table S8).

A.
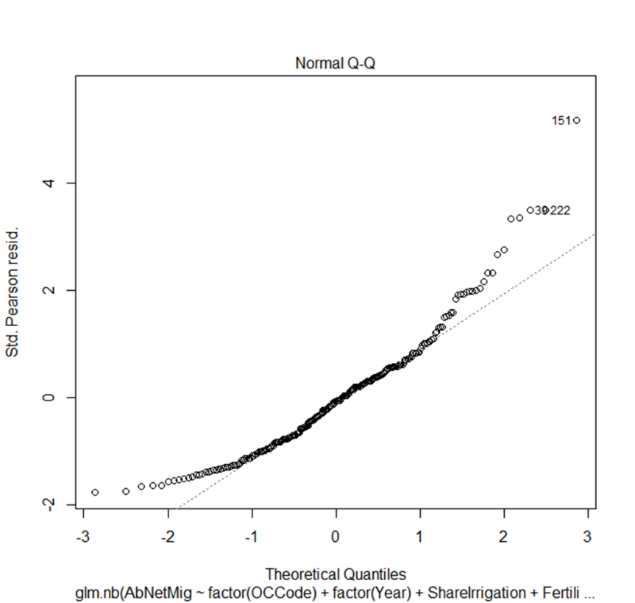
 B.
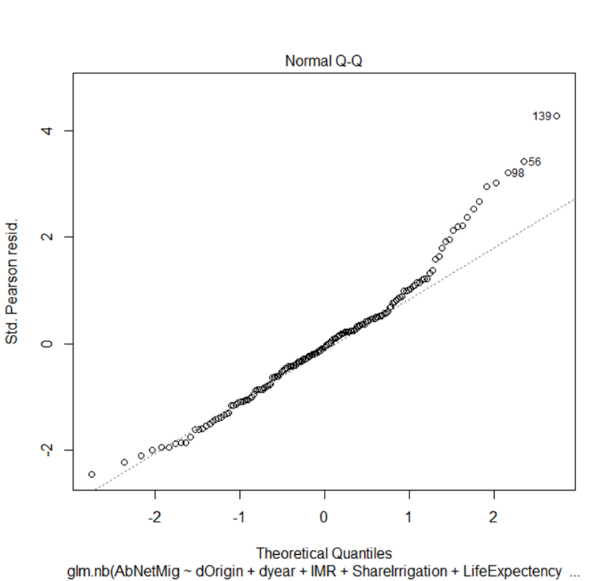


**Normal Q-Q plots for the model of international migration drivers.** **A**. Model 1: International migration from SSA countries. **B**. Model 2: International migration from emigration countries.

A.
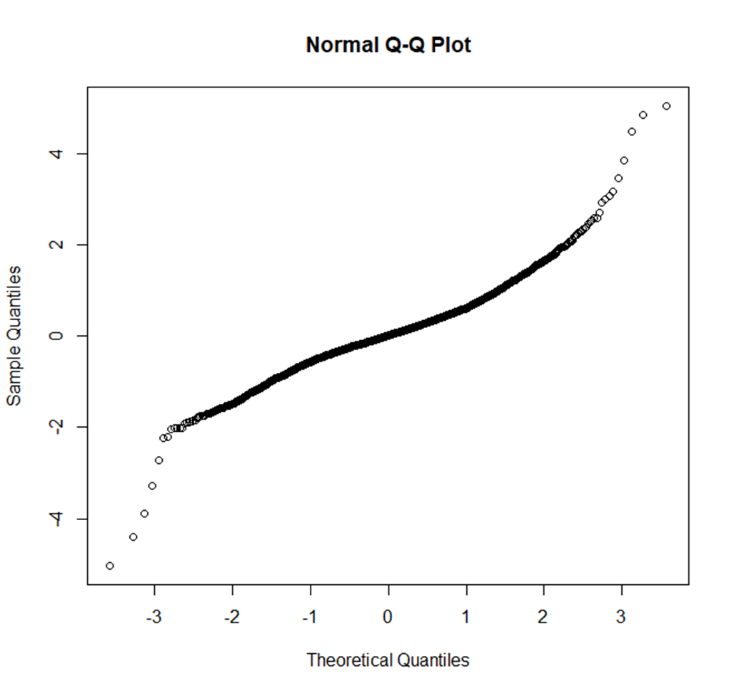
B.
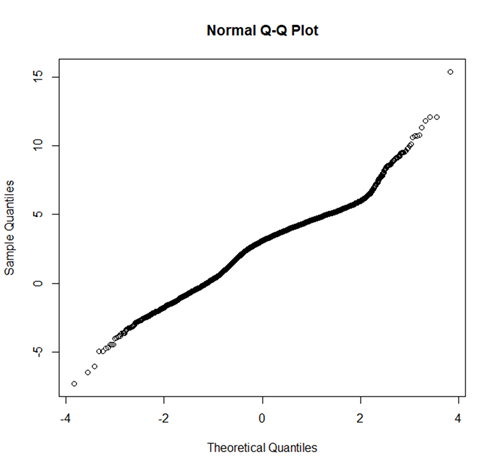
C.
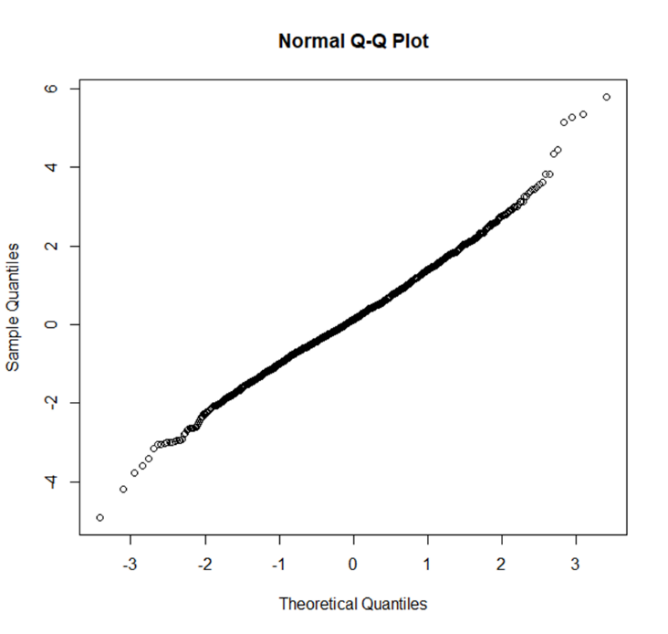


**Normal Q-Q plots for the model of cascading effects.** **A**. Expatriates in EU-14 countries. **B**. Expatriates in sub-Saharan Africa. **C**. Asylum seekers in EU-14 countries.

A.
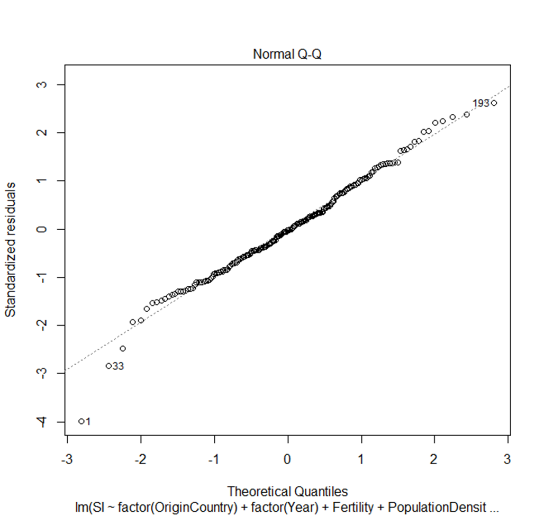
B.
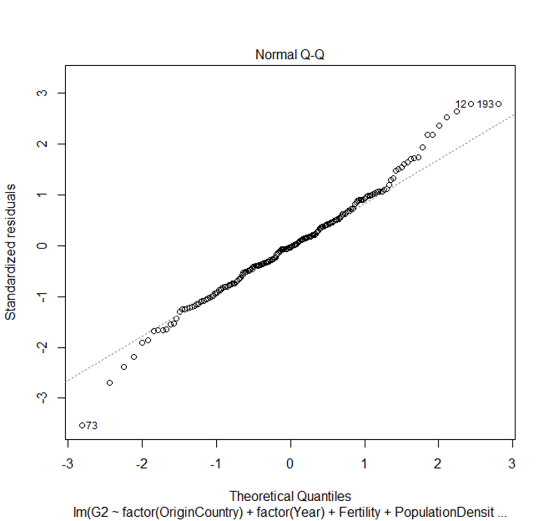
C.
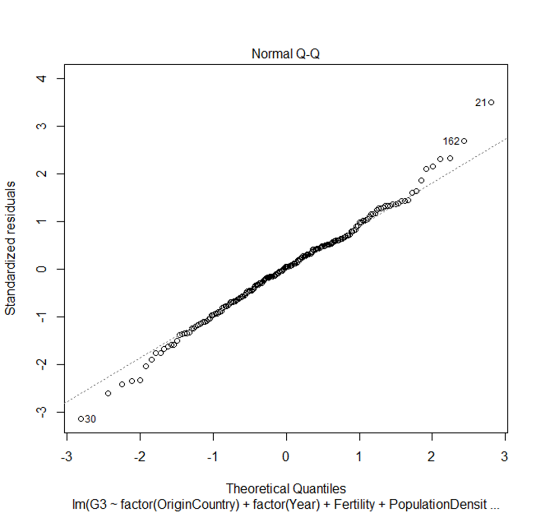
D.
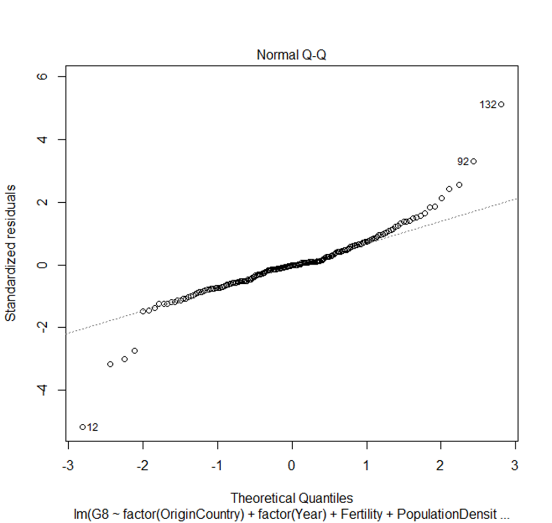


E.
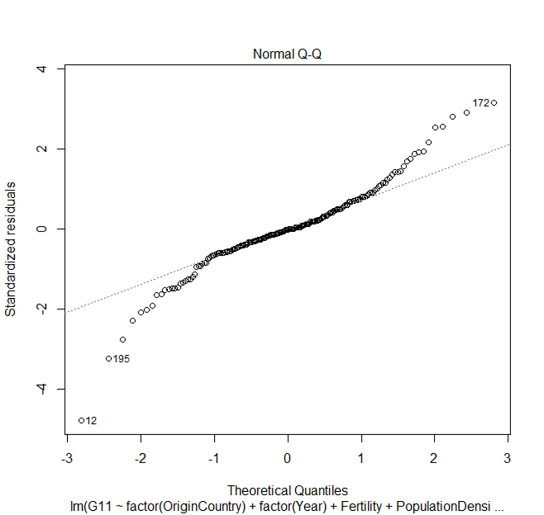
F.
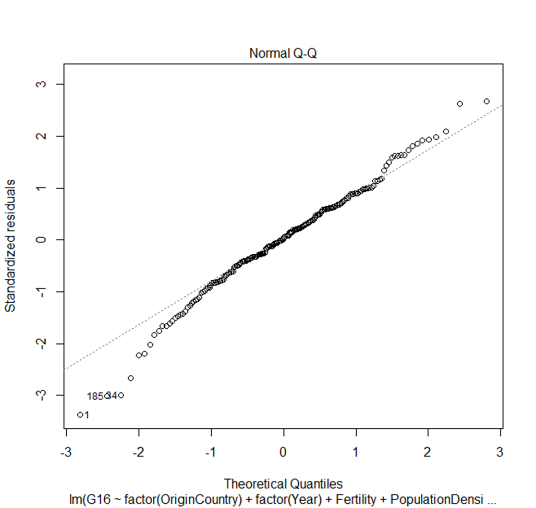


**Normal Q-Q plots for the model of feedback effects.** **A**. Sustainability: overall score. **B**. Food security and agriculture: SDG2. **C**. Healthy lives: SDG3. **D**. Sustainable economy: SDG8. **E**. Urbanization: SDG11. **F**. Peaceful societies: SDG16.

## **Robustness test**

A dataset with a reduced sample size (i.e. 85% of the observations are randomly selected) is used to test the robustness of our model of international migration drivers. In comparison to Table 2, the results of the test model shown below are consistent. This indicates the model robustness.

***Driving variables of international migration of sub-Saharan African countries***

| **Drivers** | **Variables** | **Model1:** International migration from SSA countries | | **Model2:** International migration from emigration countries | | |
| --- | --- | --- | --- | --- | --- | --- |
|  |  | Incidence rate ratio (IRR) | Coefficients | Emigration selection | Migration coefficients: | |
|  |  |  |  |  | IRR | Coefficients |
| *Demography* | Fertility | 1.2496 | 0.2228 (0.2175) | 12.2410 (3.7445) ^***^ | — | — |
|  | Population density | 0.9998 | -0.0002 (0.0034) | -0.0088 (0.0339) | — | — |
| *Climate change* | Dry extremes | 1.0001 | 0.0001 (0.0001)^*^ | -0.0001 (0.0005) | 1.0001 | 0.0001 (0.0001) ^**^ |
|  | Wet extremes | 0.9999 | -0.0001 (0.00002)^*^ | 0.0020 (0.0005) ^***^ | 0.9999 | -0.0001 (0.00003) ^***^ |
|  | Temperature extremes | 1.605 | 0.4731 (0.2766)**.** | 1.4441 (2.6478) | 0.9102 | -0.0941 (0.1816) |
| *Food security and agriculture* | Average dietary energy supply adequacy | 0.9723 | -0.0281 (0.0082)^***^ | — | 0.9958 | -0.0042 (0.0085) |
|  | Livestock production index | 1.0003 | -0.0003 (0.0048) | 0.2729 (0.0714) ^***^ | 1.0095 | 0.0094 (0.0032) |
|  | Crop production index | 0.997 | -0.0031 (0.0031) | -0.0268 (0.0410) | 0.9923 | -0.0077 (0.0029) ^***^ |
|  | Arable land per capita | 0.0494 | -3.0077 (1.3229)^*^ | — | 0.0612 | -2.7936 (1.2041)^***^ |
|  | Irrigation share | 1.0125 | 0.0125 (0.0205) | — | 1.0019 | 0.0019 (0.0214) |
| *Healthy lives* | Life expectancy | 0.9772 | -0.0231 (0.0156) | — | 0.9411 | -0.0061 (0.0135)^***^ |
| *Sustainable economy* | GDP per capita | 1 | 0.00002 (0.00004) | — | 1.0004 | 0.0004 (0.0001) ^**^ |
|  | Agro GDP share | 1.0055 | 0.0054 (0.0106) | 0.0535 (0.0762) | 0.9882 | -0.0118 (0.0105) |
|  | Unemployment rate | 1.0432 | 0.0423 (0.0278) | — | 1.0802 | 0.0771 (0.0298)^**^ |
| *Urbanization* | Urbanization rate | 1.0649 | 0.0629 (0.0185)^***^ | — | 0.9985 | 0.0015 (0.0173) |
| *Peaceful societies* | Homicide | 1.0329 | 0.0324 (0.0213) ^*^ | — | 1.0374 | 0.0367 (0.0332) |
|  | Political stability and absence of violence | 0.7281 | -0.3173 (0.1337)^*^ | — | 0.5548 | -0.5892 (0.1111)^***^ |
| *IMR* | Inverse of Mills' ratio | — | — | — | 0.5003 | -0.6925(0.2115)^**^ |
| Year fixed effect | |  | Yes | Yes | Yes | |
| Origin fixed effect | |  | Yes | Yes | Yes | |
| Constant |  | 61.3684 | 4.1169 (2.2068)**.** | -142.7400 (34.6060) ^***^ | 93.7757 | 4.5409 (1.6405) ^***^ |
| Pseudo R^2^ (Nagelkerke) | |  | 0.9789 | 0.8673 |  | 0.9996 |
| Count (N) |  |  | 200 | 200 | 142 | |

**.** , ^*^, ^**^, ^***^ = 0.1, 0.05, 0.01 and 0.001 levels of significance, respectively; figures in parenthesis indicate robust standard errors; N depicts the number of observations.

General Least Square regression is employed to test the robustness of our model of cascading effect. The below-presented results are consistent with the results derived from Tobit regression (Table S7), which shows the model robustness.

***Marginal effects of international migration on sub-Saharan African expatriates and asylum seekers***

| **Variables** | **Expatriates in:** | | **Asylum seekers in EU-14 countries** |
| --- | --- | --- | --- |
|  | EU14 | Sub-Saharan |  |
| Population density | -0.0102 (0.0068) | 0.0258 (0.0064)^***^ | 0.0089 (0.0150) |
| Distance | -1.3150 (0.2199)^***^ | -0.3040 (0.0327)^***^ | 0.4299 (0.4756) |
| Language | 0.1356 (0.0585)^*^ | 0.0609 (0.0294)^*^ | 1.1678 (0.1450)^***^ |
| Border sharing | — | 0.0843 (0.0951) | — |
| Historical migrants | 0.9011 ( 0.0123)^***^ | 0.9834 (0.0068)^***^ | 0.4132 (0.0279)^***^ |
| Migrant ratio | -1.5457 (0.8660)^.^ | -1.9003 (0.2497)^***^ | -9.9670 (1.8622)^***^ |
| Urbanization | -0.0389 (0.0217)**.** | 0.0041 (0.0100) | -0.0447 (0.0424) |
| GDP | -0.0660 (0.1752) | 0.0010 (0.0030) | 1.1457 (0.5164)^*^ |
| Emigration country | -0.0122 (0.0390) | 0.0452 (0.0374) | 0.2183 (0.1030)^*^ |
| International migration | 0.0075 (0.0161) | 0.0020 (0.0162) | 0.1866 (0.0483)^***^ |
| Year fixed effect | Yes | Yes | Yes |
| Origin fixed effect | Yes | Yes | Yes |
| Destination fixed effect | Yes | Yes | Yes |
| Adjusted R^2^ | 0.9348 | 0.9350 | 0.8035 |
| Count (N) | 2800 | 7800 | 1560 |

**.** , ^*^, ^**^, ^***^ = 0.1, 0.05, 0.01 and 0.001 levels of significance, respectively; figures in parenthesis indicate robust standard errors; McFadden's values from 0.2-0.4 indicate excellent model fit; N depicts the number of observations. The EU-14 grouping includes Austria, Belgium, Denmark, Finland, France, Germany, Greece, Republic of Ireland, Italy, Netherlands, Portugal, Spain, Sweden, and the United Kingdom.

As shown below, a dataset with a reduced sample size (i.e. 85% of the observations are randomly selected) is used to test the robustness of our model of feedback effects. The model is robust due to the consistent model output with those shown in Table S9.

***Effects of international migration on sustainability indexes***

| **Variables** | **Sustainability indexes** | | | | | | |
| --- | --- | --- | --- | --- | --- | --- | --- |
|  | **Overall score** | **Food security and agriculture: SDG2** | **Healthy lives: SDG3** | **Sustainable economy: SDG8** | **Urbanization: SDG11** | **Peaceful societies: SDG16** | |
| Fertility | -0.7267(2.2231) | -9.1121(4.6617) **.** | 2.0499(1.8213) | 1.0551(2.2916) | 0.8611(1.3129) | | -1.9629(3.0680) |
| Population density | -0.0425(0.0394) | -0.0902(0.0511) **.** | 0.0072(0.0247) | -0.0322(0.0227) | -0.0377(0.0163)^*^ | | 0.0146(0.0513) |
| Dry extremes | -0.00003(0.0004) | -0.0001(0.0004) | 0.0001(0.0003) | 0.0003(0.0002) | 0.0001(0.0001) | | -0.0006(0.0006) |
| Wet extremes | -0.0003(0.0002) | 0.0005(0.0003) **.** | -0.0001(0.0003) | 0.00003(0.0002) | -0.0002(0.0001) | | -0.0010 (0.0005) **.** |
| Temperature extremes | 0.6761(2.2680) | 0.6545(4.3840) | 6.1111(2.2436)^**^ | -5.3930(2.0911)^*^ | 1.6620(1.0543) | | -4.6017(3.1761) |
| Emigration country | -2.0876(1.3067) | -2.1184(2.1305) | 0.3907(0.9760) | -0.3620(1.1824) | 0.3879(0.6163) | | -5.4124(1.9291)^**^ |
| International migration | -0.0074(0.0033)^*^ | -0.0126(0.0068) **.** | -0.0075(0.0021)^***^ | 0.0086(0.0028)^**^ | 0.0022(0.0010)^*^ | | -0.0130(0.0046)^**^ |
| Year fixed effect | Yes | Yes | Yes | Yes | Yes | | Yes |
| Origin fixed effect | Yes | Yes | Yes | Yes | Yes | | Yes |
| Adjusted R^2^ | 0.906 | 0.572 | 0.8884 | 0.8898 | 0.9787 | | 0.7994 |
| Constant | 62.6360(17.0350)^***^ | 116.0600(34.4450)^**^ | 38.4930(13.2910)^**^ | 43.7200(18.0640)^***^ | 48.0270(10.1850)^***^ | | 80.8330(23.9910)^**^ |
| Count (N) | 160 | 160 | 160 | 160 | 160 | | 160 |

**.** , ^*^, ^**^, ^***^ = 0.1, 0.05, 0.01 and 0.001 levels of significance, respectively; figures in parenthesis indicate robust standard errors; N depicts the number of observations.

# **REFERENCES**

OECD (2015) International Migration Database, Online Edition. https://stats.oecd.org/. Accessed 1 Sep 2020

United Nations (2019) World Population Prospects 2019, Online Edition. In: Department of Economic and Social Affairs, Population Division. https://population.un.org/wpp/Download/Standard/Population/. Accessed 10 Apr 2020

United Nations (2020) International migrant stock 2019, Online Edition. In: Department of Economic and Social Affairs, Population Division, international migration. https://www.un.org/en/development/desa/population/migration/data/estimates2/estimates19.asp. Accessed 15 Apr 2020
